# Impact of timing parameters on outcomes following extracorporeal cardiopulmonary resuscitation: a systematic review and meta-analysis

**DOI:** 10.62838/jccm-2026-0049

**Published:** 2026-10-30

**Authors:** Clara Alverina, Safina Fairuz Salwaa, Muhammad Fadhil Kamaruddin, Bambang Pujo Semedi, Soni Sunarso Sulistiawan, Kun Arifi Abbas, Liong Liem

**Affiliations:** Department of Anesthesiology and Reanimation, Faculty of Medicine, Airlangga University, Surabaya, Indonesia; Department of Anesthesiology and Reanimation, Dr. Soetomo General Academic Hospital, Surabaya, Indonesia; Faculty of Medicine, Airlangga University, Surabaya, Indonesia; Graduate Institute of Biomedical Sciences-China Medical University, Taiwan 40402, R.O.C.

**Keywords:** Cardiac arrest, Cardiopulmonary resuscitation, Extracorporeal cardiopulmonary resuscitation, Outcomes, Time Intervals

## Abstract

**Background:**

Cardiac arrest remains a major public health challenge associated with high mortality. Extracorporeal cardiopulmonary resuscitation (ECPR) has emerged as a promising rescue intervention for refractory cardiac arrest; however, the association between critical time intervals and patient outcomes remains uncertain. This study examined those associations using systematic review and meta-analysis methods.

**Methods:**

A systematic review and meta-analysis was conducted per PRISMA guidelines, including 23 studies (n = 3,196) published between 2015–2024. Evaluated time intervals included no-flow duration (NFD), low-flow duration (LFD), and time from cardiac arrest to ECMO initiation (CA-to-ECMO). Outcomes assessed included neurological status using the Cerebral Performance Category (CPC) scale, survival at discharge, and hospital stay duration.

**Results:**

Patients with LFD <45 minutes showed higher survival at discharge (32% vs. 23%, p = 0.034) and more favorable neurological outcomes (26% vs. 20%, p = 0.046), though these analyses were conducted in the context of substantial between-study heterogeneity (I^2^ up to 79.7%). Meta-regression demonstrated an inverse association between LFD and survival (p = 0.024; R^2^ = 0.673) and a trend toward more favorable neurological outcomes (p = 0.057; R^2^ = 0.643). Longer NFD was associated with less favorable outcomes but without statistical significance. CA-to-ECMO time showed non-significant associations, with the most favorable results observed in our pooled data when CA-to-ECMO was <50 minutes. Stratified analyses revealed shorter LFD thresholds were associated with more favorable outcomes for IHCA, while OHCA outcomes declined markedly when LFD exceeded 70 minutes.

**Conclusions:**

LFD was associated with outcomes in ECPR, the most favorable outcomes were observed when LFD was less than 45 minutes. These findings should be interpreted as hypothesis-generating given the observational study designs and potential for residual confounding. Location-specific timing thresholds and rapid ECMO deployment may contribute to more favorable outcomes but require prospective validation before operational recommendations can be made.

## Introduction

Cardiac arrest continues to represent a major public health challenge. In the United States alone, over 356,000 out-of-hospital cardiac arrests (OHCA) occur annually, with nearly 90% resulting in death [1]. Additionally, the incidence of in-hospital cardiac arrest (IHCA) has been reported to be rising across several countries [2]. Conventional cardiopulmonary resuscitation (CCPR) as the standard initial intervention, within optimal conditions, achieves only 20–30% of normal cardiac output [3].

When CCPR fails to restore spontaneous circulation within 10–15 minutes, extracorporeal cardiopulmonary resuscitation (ECPR) may be considered for both IHCA and OHCA cases [4,5]. By providing superior systemic perfusion compared to CCPR, ECPR has the potential to limit anoxic injury and reduce ischemia–reperfusion damage in post-cardiac arrest patients [6,7].

Several studies recommend initiating ECPR within 60 minutes of CCPR initiation [8,9]. Previous studies also demonstrated improved neurological outcomes, shorter hospital stays, and increased survival in patients who receive ECPR following shorter durations of low-flow—the period during which CCPR is ongoing but before ECMO support is established [6,10, 11, 12, 13, 14]. Despite growing evidence on the utility of ECPR, consensus on the optimal time thresholds remains lacking. Therefore, this systematic review and meta-analysis aims to explore whether specific timing ranges are associated with outcomes following ECPR.

## Materials and methods

### Study Design

This systematic review and meta-analysis were conducted in accordance with the Preferred Reporting Items for Systematic Reviews and Meta-Analyses (PRISMA) guidelines. The protocol was registered in PROSPERO (registration number: [PROSPERO ID CRD420251037420]). This study is based on previously published research and does not involve new human or animal subjects. Therefore, ethical approval and informed consent were not required.

### Search Strategy

A comprehensive literature search was performed in multiple electronic databases, including Cochrane Library, EBSCO, PubMed, Science Direct, and Scopus. The search covered studies published between January 2015 and December 2024. The search strategy combined Medical Subject Headings (MeSH) terms and keywords related to extracorporeal cardiopulmonary resuscitation, extracorporeal membrane oxygenation, cardiac arrest, and outcomes.

### Study Selection Process and Selection Criteria

The study selection process was conducted in two phases. Initially, two independent reviewers screened titles and abstracts of all retrieved articles to identify potentially eligible studies. Full texts of the selected articles were then assessed against the predefined inclusion and exclusion criteria to ensure consistency in population, intervention, timing variables, and outcome measures. Discrepancies were resolved through consensus with a third reviewer. Studies of patients with traumatic cardiac arrest and post-cardiotomy were excluded a priori because their pathophysiology (haemorrhagic and surgical) differs fundamentally from the ischaemic mechanism underlying non-traumatic refractory cardiac arrest, and ECPR indications and expected outcomes differ substantially. This exclusion improves the clinical homogeneity of the analytic population and aligns our synthesis with the populations for which ECPR guideline recommendations are currently framed. The criteria applied during review are summarized in Table 1.

**Table 1.** Studies were selected based on predefined inclusion and exclusion criteria

**Inclusion Criteria**	**Exclusion criteria**
● Adult patients (≥18 years of age)	● Patients who underwent cardiac surgery
● Patients resuscitated from in-hospital or out-of-hospital cardiac arrest	● Publications such as letters, opinions, case reports, case series, review articles, meta-analyses
● Enrolled patients receiving ECPR with or without emergency coronary angiography (CAG) or percutaneous coronary intervention (PCI)	● Studies conducted on pediatric populations, pregnancy, presumed pregnancy, or patients with a pulse (e.g., cardiogenic shock)
● Studies published between January 2015 and December 2024	● Patients with Do Not Resuscitate (DNR) status
● Studies reporting at least one timing data point from: No Flow Duration (NFD) or Low Flow Duration (LFD) or CA-to-ECMO Duration	● Cardiac arrest of traumatic origin
● Studies reporting at least one of the following outcomes: hospital length of stay (LOS) / survival / neurological outcomes	● Studies using definitions of timing predictors and outcomes different from those established in this study
● Studies using the Cerebral Performance Category (CPC) scale as criteria for favorable / unfavorable neurological outcomes	● Studies that do not clearly describe their timing parameter definitions
● Studies published in English as full-text articles in indexed journals	

### Data Extraction

Data extraction was performed independently by three reviewers using a standardized form. The following information was extracted from each included study: first author, year of publication, country, study design, sample size, patient demographics (age, gender), comorbidities, cardiac arrest characteristics (IHCA or OHCA, initial rhythm, etiology), timing parameters (NFD, LFD, CA-to-ECMO Duration), extracorporeal membrane oxygenation (ECMO) procedure details, and outcome measures (hospital LOS, neurological outcomes using CPC scale, and survival at discharge).

### Definition of Key Variables

#### Predictors

The primary predictors of interest were time-to-ECMO parameters. To ensure methodological consistency, only studies with equivalent timing definitions (i.e., comparable start and end anchors) were included in the quantitative synthesis. Timing variables were defined according to physiologic flow states and analyzed as separate predictors to avoid misclassification across heterogeneous definitions. When multiple timing metrics were reported within a study, the measure most consistent with the predefined physiologic construct was selected according to the registered protocol. A full per-study mapping of the timing definitions used in each included study, together with the harmonization decisions applied when reporting differed, is provided in Supplementary Table S1.

Timing predictors were defined as follows:

- No Flow Duration (NFD): The time interval between the onset of cardiac arrest and the initiation of cardiopulmonary resuscitation (CPR), representing the period when there is a complete absence of blood flow to vital organs, also commonly known as “collapse-to-CPR time” or “arrest-to-CPR time.”
- Low Flow Duration (LFD): The time interval between the initiation of CPR and the establishment of ECMO flow, representing the period when blood flow is maintained solely through manual or mechanical chest compressions, also known as “CPR-to-ECMO time.”
- Cardiac Arrest to ECMO Duration: The total elapsed time from the onset of cardiac arrest to the successful establishment of ECMO flow, calculated as the sum of NFD and LFD, also referred to as “collapse-to-ECMO time” or “arrest-to-ECMO time.”

Additional clinical predictors included: patient age and gender, cardiac arrest location (IHCA/OHCA/both), comorbidities, initial shockable cardiac rhythm and cardiac etiology.

#### Outcomes

- Neurological Outcomes: Assessed using the Cerebral Performance Category (CPC) scale. For this study, CPC scores of 1–2 were considered favorable neurological outcomes, while scores of 3–5 were considered unfavorable.
- Survival at Discharge: The state of a patient remaining alive following cardiac arrest and subsequent ECPR intervention, assessed at hospital discharge, with patients categorized as either survivors or non-survivors based on their vital status at the defined assessment point.
- Hospital Length of Stay (LOS): The total duration, measured in days, that a patient remains admitted in the hospital from the time of admission following cardiac arrest and ECPR intervention until discharge, transfer to another facility, or death, representing a key metric for evaluating healthcare resource utilization and recovery trajectory.

### Statistical Analysis

Data analysis was performed using R-Studio version 4.5.3. Statistical heterogeneity among studies was assessed using Cochran’s Q test and the I^2^ statistic, with I^2^ values of 25%, 50%, and 75% representing low, moderate, and high heterogeneity, respectively. Given the anticipated clinical and methodological heterogeneity across the included observational studies, all pooled analyses were performed using the DerSimonian–Laird random-effects model regardless of the observed level of statistical heterogeneity. For continuous variables such as NFD, LFD, CA-to-ECMO duration, and hospital length of stay (LOS), pooled mean differences (MDs) with corresponding 95% confidence intervals (CIs) were calculated. For dichotomous outcomes, including favorable neurological outcome (CPC 1–2) and survival at hospital discharge, pooled proportions with corresponding 95% confidence intervals were calculated.

The primary analysis for each timing parameter was a meta-regression treating the timing variable as a continuous predictor, with the corresponding outcome proportion (favorable neurological outcome or survival at discharge) as the dependent variable. This approach was chosen to avoid the loss of information and risk of spurious threshold effects that accompany dichotomization. Dichotomized comparisons at pre-specified cutoffs (LFD <45 vs ≥45 minutes; CA-to-ECMO <50 vs ≥50 minutes; NFD <3 vs ≥3 minutes) were retained as secondary, descriptive analyses intended to aid clinical interpretation. The 45-minute LFD cutoff was chosen based on the distribution of reported LFD values in the included studies and for alignment with prior observational literature (Park et al. 2020 [15]; Ohbe et al. 2022 [16]) and the intermediate position between the European Resuscitation Council recommendation of <60 minutes [5] and the Japanese target of <40 minutes [17]; we acknowledge that the cutoff is data-driven rather than derived from randomized evidence.

For specific comparisons (e.g., LFD <45 minutes versus ≥45 minutes), odds ratios (OR) with 95% confidence intervals (CI) were computed to quantify the magnitude of effect. Meta-regression analyses were conducted to examine relationships between timing parameters and outcomes, with R^2^ values reported to indicate the strength of these associations.

Subgroup analyses were performed as primary stratified analyses based on the type of cardiac arrest (IHCA vs. OHCA), recognizing that these populations differ in response times, witness status, and system-level care pathways. Pooled IHCA+OHCA estimates are reported for completeness but should be interpreted with caution given the underlying clinical heterogeneity. Sensitivity analyses were conducted by sequentially excluding individual studies to evaluate their influence on the pooled estimates. Data visualization and graphing were performed using GraphPad Prism 10.4.1.

### Handling of Missing Data

For each analysis, studies that did not report the required timing variable or outcome were excluded from that specific analysis (pairwise deletion); no imputation was performed for missing outcomes. When studies reported central tendency as median with interquartile range or range rather than mean with standard deviation, values were converted using the method of Wan et al. (2014), which estimates the sample mean and standard deviation from the sample size, median, and IQR; detailed scenario assignment (Wan Scenario C1 for median + range; C2 for median + Q1 + Q3) and the special-case reconstruction for Wengenmayer (2017, mean ± 95% CI) are documented in Supplementary Methods S4. We recognize that pairwise deletion can introduce bias if missingness is not completely at random, and we address this explicitly in the Limitations section. Corresponding authors of included studies were not contacted for missing timing data; this potential limitation is acknowledged in the Limitations section.

### Sensitivity Analyses and Assessment of Publication Bias

To assess the robustness of our pooled estimates, we performed leave-one-out sensitivity analyses in which each included study was sequentially excluded and the pooled estimate recomputed, examining whether the direction, magnitude, or statistical significance of the effect was influenced by any single study. Pooled estimates remained stable across leave-one-out iterations; full results are provided in Supplementary Table S5 and Supplementary Figure S6. For outcomes informed by ten or more studies, we assessed small-study effects and potential publication bias using visual inspection of funnel plots (Supplementary Figure S5) and Egger’s regression test. For outcomes informed by fewer than ten studies, formal tests for publication bias were not performed because of insufficient statistical power. Among all pooled estimates, only the OHCA CA-to-ECMO × favorable neurological outcome subgroup met the k ≥10 threshold for formal Egger testing; the Egger test yielded p = 0.414, providing no statistical evidence of small-study asymmetry. Full Egger regression statistics for all outcomes are provided in Supplementary Table S6. Given the absence of funnel plot asymmetry in this eligible outcome and the visual symmetry observed across other outcomes, trim-and-fill correction was not applied. For all other outcomes, publication bias cannot be formally excluded.

### Certainty of Evidence (GRADE)

The certainty of evidence for each primary outcome (favorable neurological outcome and survival at discharge, stratified by timing parameter) was assessed using the Grading of Recommendations, Assessment, Development and Evaluations (GRADE) framework, considering the five domains of risk of bias, inconsistency, indirectness, imprecision, and publication bias. Because all included studies were observational, the starting certainty was low, and further downgrading was applied where warranted. A Summary of Findings table is presented in Supplementary Table S2.

## Results

### Study Selection

We identified 3,196 studies through database searching. After removing duplicates and screening process, 23 studies met the inclusion criteria and were incorporated into the review [Figure 1].

**Figure 1.**
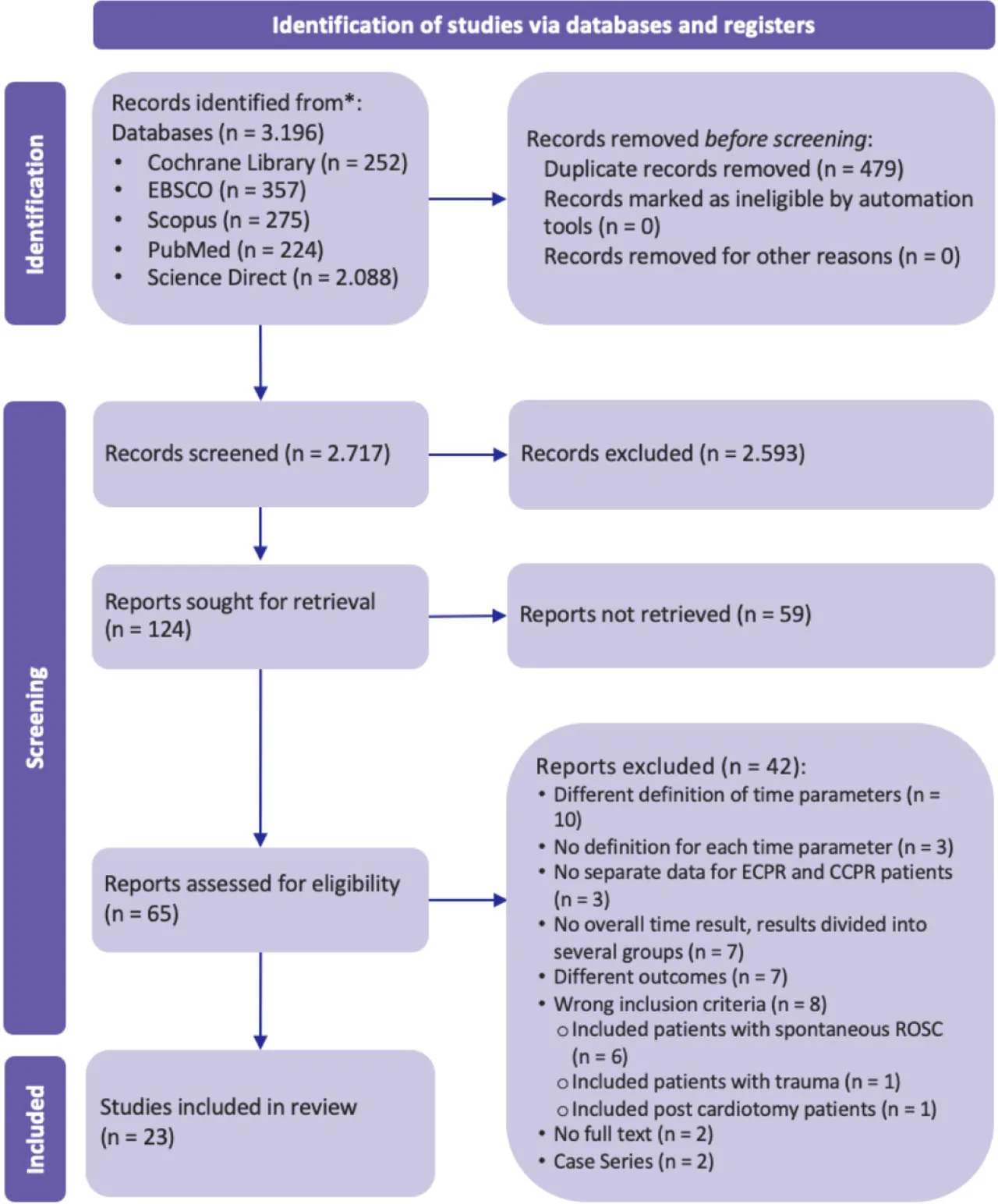
PRISMA 2020 flow diagram for new systematic reviews, which included searches of databases, registers, and other sources.

### Risk of Bias and Quality Assessment

The risk of bias and study quality were independently evaluated by two reviewers using the Cochrane ROB-2 tool for randomized trials [18] and ROBINS-I tool [19] for non-randomized studies. Among the 23 included studies, most observational designs showed moderate bias, mainly from confounding and participant selection, while two [20,21] had serious risk [Supplementary Figure 4a]. Recent studies were generally lower risk, and both RCTs [22,23] showed some concerns due to deviations and missing data [Supplementary Figure 4b]. Overall, methodological quality has improved, but residual confounding and selection bias remain important limitations.

### Study Characteristics and Population

A total of 23 studies, published between 2017 and 2024, were included in the review with sample sizes ranging from 14 to 482 patients. The studies adopted a retrospective design (n = 19), prospective observational study (n = 2), a randomized clinical trial (n = 1), and a pre-planned protocol analysis of a multicenter randomized trial (n = 1). Eleven studies included exclusively OHCA patients, five focused on IHCA, and seven reported mixed populations [Table 2].

**Table 2.** Study Characteristics

**No**	**First Author**	**Year**	**Country**	**Study Design**	**Setting (SC/MC)**	**Sample Size, n**	**CA Location, n (%)**
1	Cesana* [46]	2017	Italy	retrospective study	SC	63	IHCA 17 (27); OHCA 46 (73)
2	Dennis* [47]	2017	Australia	retrospective analysis study	MC	37	IHCA 25 (76); OHCA 12 (24)
3	Elmelliti [60]	2023	Qatar	retrospective cohort study	SC	48	IHCA 11 (22.9); OHCA 37 (77.1)
4	Fjølner* [20]	2017	Denmark	retrospective cohort study	SC	21	OHCA 21 (100)
5	Fu [61]	2024	Taiwan	retrospective cohort study	SC	14	OHCA 14 (100)
6	Gaisendrees [62]	2024	Germany	retrospective cohort study	SC	157	IHCA 66 (42); OHCA 91 (58)
7	Higashi [42]	2020	Japan	retrospective observational study	SC	117	IHCA 117 (100)
8	Murakami* [33]	2020	Japan	retrospective observational study	SC	85	OHCA 85 (100)
9	Ng [63]	2022	Hong Kong	retrospective study	SC	102	IHCA 54 (52.9); OHCA 48 (47.1)
10	Otani* [34]	2018	Japan	retrospective analysis study	SC	135	OHCA 135 (100)
11	Podell [21]	2022	USA	prospective observational study	SC	57	IHCA 52 (91); OHCA 5 (9)
12	Ryu [64]	2019	South Korea	retrospective observational study	SC	274	IHCA 250 (91.24); OHCA 24 (8.76)
13	Shih* [35]	2024	Taiwan	retrospective cohort study	SC	85	OHCA 85 (100)
14	Stub [65] (CHEER)	2015	Australia	prospective, observational trial	SC	26	IHCA 15 (57.7); OHCA 11 (42.3)
15	Takahagi* [37]	2022	Japan	retrospective consecutive study	SC	39	OHCA 39 (100)
16	Ubben* [23] (INCEPTION-trial)	2023	Netherlands	pre-planned, per-protocol, randomized controlled trial	MC	33	OHCA 33 (100)
17	Vahedian-Azimi [66]	2024	Qatar	retrospective observational study	SC	48	IHCA 11 (22.9); OHCA 37 (77.1)
18	Wengenmayer [43]	2017	Germany	retrospective registry data analysis	SC	133	IHCA 74 (55.6); OHCA 59 (44.4)
19	Yu [67]	2019	Taiwan	prospective observational study	SC	482	IHCA 377 (78.2); OHCA 105 (21.8)
20	Yukawa* [31]	2017	Japan	retrospective study	SC	79	OHCA 79 (100)
21	Puolakka [68]	2023	Finland	retrospective observational cohort study	SC	37	OHCA 37 (100)
22	Ellouze [69]	2017	France	retrospective observational study	SC	65	IHCA 43 (66.2); OHCA 22 (33.8)
23	Rob* [22] (Prague OHCA trial)	2022	Czech Republic	randomized controlled trial	SC	92	OHCA 92 (100)

* Some of the samples underwent PCI/CAG preceding or following the ECPR procedure.

N, number of samples; NR, not reported; SC: single-center; MC: multi-center; OHCA: out-of-hospital cardiac arrest; IHCA: in-hospital cardiac arrest.

Patient age ranged from the early 40s to the late 60s, with most cohorts reporting mean or median ages between 50 and 60 years. The majority of patients were male. Comorbidity data, although variably reported, most frequently included hypertension, diabetes mellitus, dyslipidemia, and coronary artery disease. Cardiac arrest etiology was reported inconsistently, but when available, the predominant cause was of cardiac origin, with proportions ranging from 36% to 100% [Table 3].

**Table 3.** Population Characteristics

**No**	**First Author**	Age, year **	Male*	Comorbidities*	Cardiac Etiology*	Shockable Initial Rhythm*
1	Cesana [46]	59±10	55 (87.3)	HTN 31 (49); DM 8 (13); Dyslipidemia 5 (9); CKD 2 (3); CAD 8 (13)	63 (100)	41 (65.1)
2	Dennis [47]	54 (47–58)	27 (73)	HTN 18 (49); DM 12 (32); Dyslipidemia 14 (38); CAD 9 (24)	14 (37.8)	19 (51.35)
3	Elmelliti [60]	41.88±11.5	36 (75)	HTN 7 (14.6); DM 10 (20.8); Dyslipidemia 3 (6.3); CAD 6 (12.5); HF 1 (2.1)	21 (43.8)	16 (33.3)
4	Fjølner [20]	56 (19–73)	12 (57)	NR	18 (85.7)	9 (43)
5	Fu [61]	54 (46.5–61.8)	13 (92.9)	NR	NR	11 (78.6)
6	Gaisendrees [62]	55.7±12.8	126 (80.3)	NR	NR	93 (59.5)
7	Higashi [42]	66 (46–75)	77 (65.8)	NR	80 (68.4)	29 (25.7)
8	Murakami [33]	56.7±11.6	70 (82.4)	NR	61 (71.7)	70 (82.4)
9	Ng [63]	54 (45–61)	73 (71.6)	NR	74 (72.5)	53 (52)
10	Otani [34]	65 (50–72)	115 (85)	NR	92 (68.1)	87 (64)
11	Podell [21]	57 (IQR:18)	37 (65)	NR	NR	21 (36.8)
12	Ryu [64]	62.7 (47–74)	174 (63.5)	HTN 123 (44.9); DM 90 (32.8); Dyslipidemia 32 (11.6); CKD 40 (14.6); CAD 39 (14.2)	114 (41.6)	79 (28.83)
13	Shih [35]	56 (45–64)	71 (83.5)	HTN 25 (29.4); DM 23 (27.1); CAD 14 (16.5); CHF 2 (2.4); CVA 4 (4.7); CKD 6 (7.1); Cancer 1 (1.2)	NR	74 (87.1)
14	Stub [65]	52 (38–60)	20 (76.9)	HTN 11 (42); DM 2 (8); Dyslipidemia 11 (42) CHF 5 (19); CAD 4 (15)	22 (84.6)	19 (73.07)
15	Takahagi [37]	66 (48–72)	26 (66.7)	NR	14 (35.6)	0
16	Ubben [23]	55.0±11.0	30 (91)	ACS 5 (18); CAD 4 (14); HF 1 (3); CVA 2 (7); DM 4 (14); HTN 11 (55); Dyslipidemia 5 (28)	25 (75.7)	33 (100)
17	Vahedian-Azimi [66]	41.88±11.5	36 (75)	HTN 7 (14.6); DM 10 (20.8); Dyslipidemia 3 (6.3); CAD 6 (12.5); HF 1 (2.1)	21 (43.8)	16 (33.3)
18	Wengenmayer [43]	58.7±2.6	99 (74.4)	NR	NR	76 (57.1)
19	Yu [67]	56.4±15.1	368 (76.3)	DM 176 (36.5); CAD 190 (39.4); CKD 71 (14.7)	268 (55.6)	NR
20	Yukawa [31]	59 (48.5–64.5)	65 (82.3)	NR	31 (39.2)	58 (73.4)
21	Puolakka [68]	51.9±12.1	34 (91.9)	NR	NR	29 (78.4)
22	Ellouze [69]	56 (43–65)	45 (69.2)	NR	48 (73.9)	22 (33.8)
23	Rob* [22]	58.5 (45–65)	76 (82.6)	HTN 38 (46.3); DM 13 (16.3); COPD 2 (2.5); CKD 2 (2.5); CAD 15 (18.8); HF 9 (11.1)	NR	57 (61.9)

* data presented as n(%)

** data presented as mean±SD or median(IQR)

N, number of samples; NR, not reported; DM, diabetes mellitus; HTN, hypertension; ACS: acute coronary syndrome; CAD: coronary artery disease; CVD, cardiovascular disease; CKD, chronic kidney disease; HF: heart failure; CVA: cerebrovascular accident; PAD: peripheral artery disease.

### Timing Parameters

NFD was reported in 8 of the 23 included studies as a continuous numeric variable, with mean/median values ranging from 0 to 6.31 minutes (4 additional studies recorded NFD = 0 as a definitional value rather than a measured timestamp). A comparison of baseline and design characteristics between the 8 NFD-reporting studies and the 15 that did not report continuous NFD is provided in Supplementary Table S3. Both groups were broadly comparable in median sample size and single-center prevalence; notably, NFD-non-reporting studies were dominated by OHCA-only designs (60%) — consistent with the difficulty of capturing collapse-to-CPR times in the pre-hospital setting — whereas NFD-reporting studies more frequently enrolled mixed IHCA/OHCA cohorts. No clear temporal trend in NFD reporting was observed (median publication year 2020 vs. 2019 for non-reporters).

IHCA studies consistently reported minimal NFD (0–0.3 minutes), while OHCA studies reported longer durations (mean/median 3.0–5.4 minutes). LFD was reported in 14 studies, with mean/median values ranging from 27 to 72.2 minutes. IHCA studies generally demonstrated shorter LFD (mean/median 27–49.6 minutes) compared to OHCA studies (mean/median 47–72.2 minutes). CA-to-ECMO duration was reported in 14 studies, with mean/median ranging from 31 to 121 minutes. IHCA studies consistently showed shorter durations (mean/median 40–45 minutes) compared to OHCA studies (mean/median 44–121 minutes) [Table 4 – 6].

**Table 4.** Timing and Outcomes of Both OHCA & IHCA groups

**No**	**First Author**	**Sample Size (n)**	**Timing**			**Outcomes**			
			No-flow duration, min **	Low-flow duration, min**	CA-to-ECMO, min**	Favorable outcome*	Survival at 30 days*	Survival at discharge*	Hospital LOS, day**
1	Ryu [64]	274	NR	32.2 (13.0–57.0)	**NR**	78 (28.5%)	NR	95 (34.7%)	NR
2	Siao [36]	20	1 – 5	34.25 (15–226)	NR	8 (40%)	NR	10 (50%)	NR
3	Yu [67]	482	NR	40.8 ± 21.4	NR	126 (26.1%)	NR	143 (29.6%)	NR
4	Gaisendrees [62]	157	3.25 ± 4.9	56.08 ± 28.0	NR	NR	NR	39 (24.8%)	11.4 ± 19.5
5	Wengenmayer [43]	133	2.6 ± 0.8	59.6 ± 5.0	NR	NR	NR	19 (14.3%)	NR
6	Vahedian-Azimi [66]	48	6.31 ± 3.37	39.5 ± 19.63	45.81 ± 20.23	9 (18.8%)	14 (29.2%)	NR	4 (1–12)
7	Cesana [46]	63	3.0 ± 4.0	56.0 ± 24.0	58.0 ± 25.0	12 (19.04%)	NR	13 (21%)	68.0 ± 33.0
8	Ng [63]	102	0 (0–1)	52 (40–73)	56 (41–74)	19 (18.6%)	25 (24.5%)	33 (32.4%)	7 (2–30)
9	Ellouze [69]	65	0 (0–0)	70 (45–110)	NR	15 (23.1%)	NR	16 (24.6%)	NR
10	Podell [21]	57	NR	NR	31 (23)	23 (40.3%)	NR	26 (45.6%)	22.0 ± 18.52
11	Dennis [47]	37	NR	NR	45 (30–70)	13 (35.2%)	NR	13 (35.2%)	11 (2–34)
12	Elmelliti [60]	48	NR	NR	45 (30–59.5)	10 (20.8%)	NR	10 (20.8%)	4 (1–12)
13	Stub [65]	26	NR	NR	56 (40–85)	14 (54%)	NR	14 (54%)	NR

* data presented as n(%)

** data presented as mean±SD or median(IQR)

**Table 5.** Timing and outcomes of OHCA only group

**No**	**First Author**	**Sample Size (n)**	**Timing**			**Outcomes**			
			No-flow duration, min **	Low-flow duration, min**	CA-to-ECMO, min**	Favourable outcome*	Survival at 30 days*	Survival at discharge*	Hospital LOS, day**
1	Otani [34]	135	NR	47 (41–57)	NR	22 (16.3%)	NR	34 (25%)	NR
2	Gaisendrees [62]	91	4.3 ± 5.2	63.6 ± 25.1	NR	NR	NR	21 (23.1%)	6.7 ± 16.2
3	Wengenmayer [43]	59	5.4 ± 1.5	72.2 ± 7.4	NR	NR	NR	5 (8.5%)	NR
4	Murakami [33]	85	4.6 ± 5.6	49.6 ± 13.4	54.2±14.6	14 (16%)	NR	NR	NR
5	Ellouze	22	0 (0–5)	90 (66–138)	NR	6 (27.3%)	NR	6 (27.3%)	NR
6	Fjølner [20]	21	NR	NR	121 (55–192)	7 (33.3%)	NR	7 (33.3%)	NR
7	Fu [61]	14	NR	NR	48.5 (43.8–56.2)	4 (28%)	NR	5 (35%)	NR
8	Dennis [47]	12	NR	NR	63 (45–77)	5 (41%)	NR	5 (41%)	NR
9	Shih [35]	85	NR	NR	92.0 (76.0–118. 0)	15 (17.6%)	NR	25 (29.4%)	NR
10	Takahagi [37]	39	NR	NR	44 (35–55)	4 (10.3%)	7 (18%)	NR	NR
11	Ubben [23]	33	NR	NR	69 (60–77)	5 (15%)	5 (15%)	5 (15%)	2 (2–15)
12	Yukawa [31]	79	NR	NR	45.0 (40.0–56.5)	11 (13.9%)	NR	NR	NR
13	Puolakka [68]	37	NR	NR	84 (61–105)	11 (29.7%)	NR	13 (35.1%)	NR
14	Rob [22]	92	NR	NR	61 (55–70)	20 (21.7%)	NR	NR	NR

* data presented as n(%)

** data presented as mean±SD or median(IQR)

**Table 6.** Timing and outcomes of IHCA only group

**No**	**First Author**	**Sample Size (n)**	**Timing**			**Outcomes**			
			No-flow duration, min**	Low-flow duration, min**	CA-to-ECMO, min**	Favourable outcome*	Survival at 30 days*	Survival at discharge*	Hospital LOS, day**
1	Higashi [42]	117	0	27 (19–40)	NR	37 (31.9%)	NR	45 (38.8%)	10 (4–19)
2	Gaisendrees [62]	66	0	41.1 ± 27.4	NR	18 (27.27%)	NR	NR	15.6 ± 7.7
3	Wengenmayer [43]	74	0.3 ± 0.3	49.6 ± 5.9	NR	NR	NR	14 (18.9%)	NR
4	Ellouze [69]	43	0	60 (45–89)	NR	9 (20.9%)	NR	10 (23.3%)	NR
5	Dennis [47]	25	NR	NR	40 (30–55)	8 (33%)	NR	8 (33%)	NR

* data presented as n(%)

** data presented as mean±SD or median(IQR

### Meta-Analysis Results

Full meta-regression coefficients (β, p, R^2^) for all 16 stratum × outcome combinations are reported in Supplementary Table S7; verification of all primary BOTH-stratum analyses against an independent re-run in R is provided in Supplementary Table S8.

### No-flow duration

Explanatory analyses of NFD showed no significant association with neurological or survival outcomes. However, interpretation was limited by the small number of contributing studies, resulting in low statistical power and limited robustness of pooled estimates [Supplementary Figure 1a] [Supplementary Figure 2c].

### Low-flow duration

Our findings demonstrated that longer LFDs were consistently associated with lower rates of favorable neurological recovery [Figure 2] and survival at discharge [Figure 3]. In the primary meta-regression, LFD treated as a continuous predictor was inversely associated with survival at discharge (β = −0.564; p = 0.024; R^2^ = 0.673) and with favorable neurological outcome (β = −0.466; p = 0.057; R^2^ = 0.643), with outcomes declining progressively across the observed range rather than in a sharp threshold fashion. In the secondary dichotomized analysis, LFD <45 minutes was associated with higher favorable neurological outcome than LFD ≥45 minutes (p = 0.046; pooled I^2^ = 32.7%). Subgroup analysis of OHCA- and IHCA-only populations further confirmed this trend, with low heterogeneity across studies (I^2^ = 0% and 1.4% respectively) [Figure 4].

**Figure 2.**
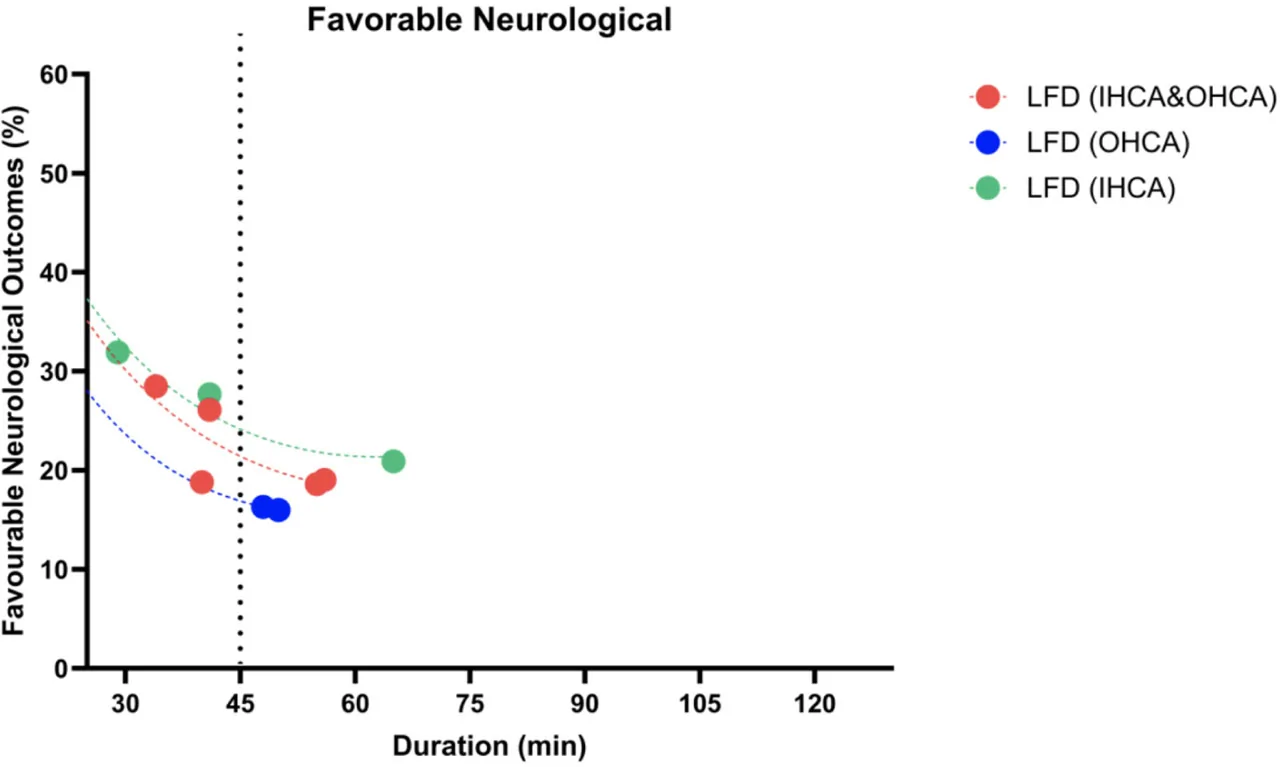
Relation between LFD and Neurological Outcomes

**Figure 3.**
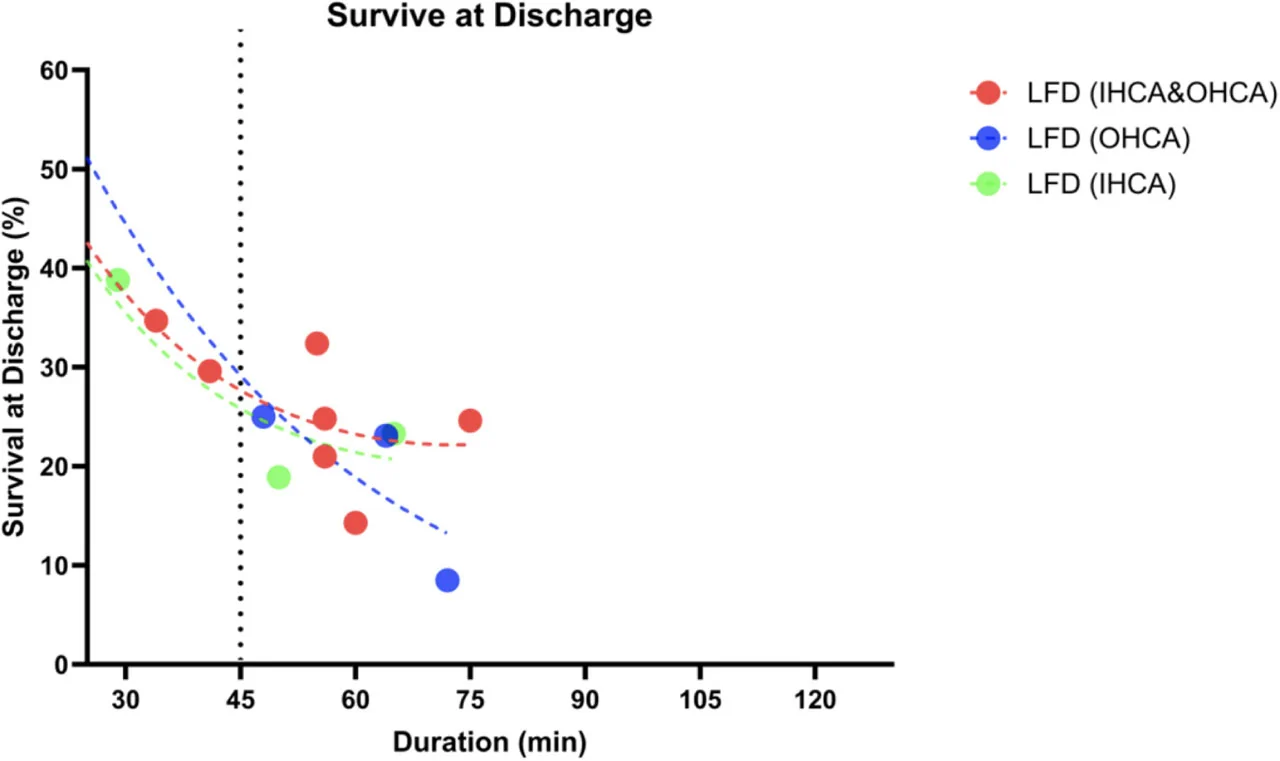
Relation between LFD and Survival at Discharge

**Figure 4.**
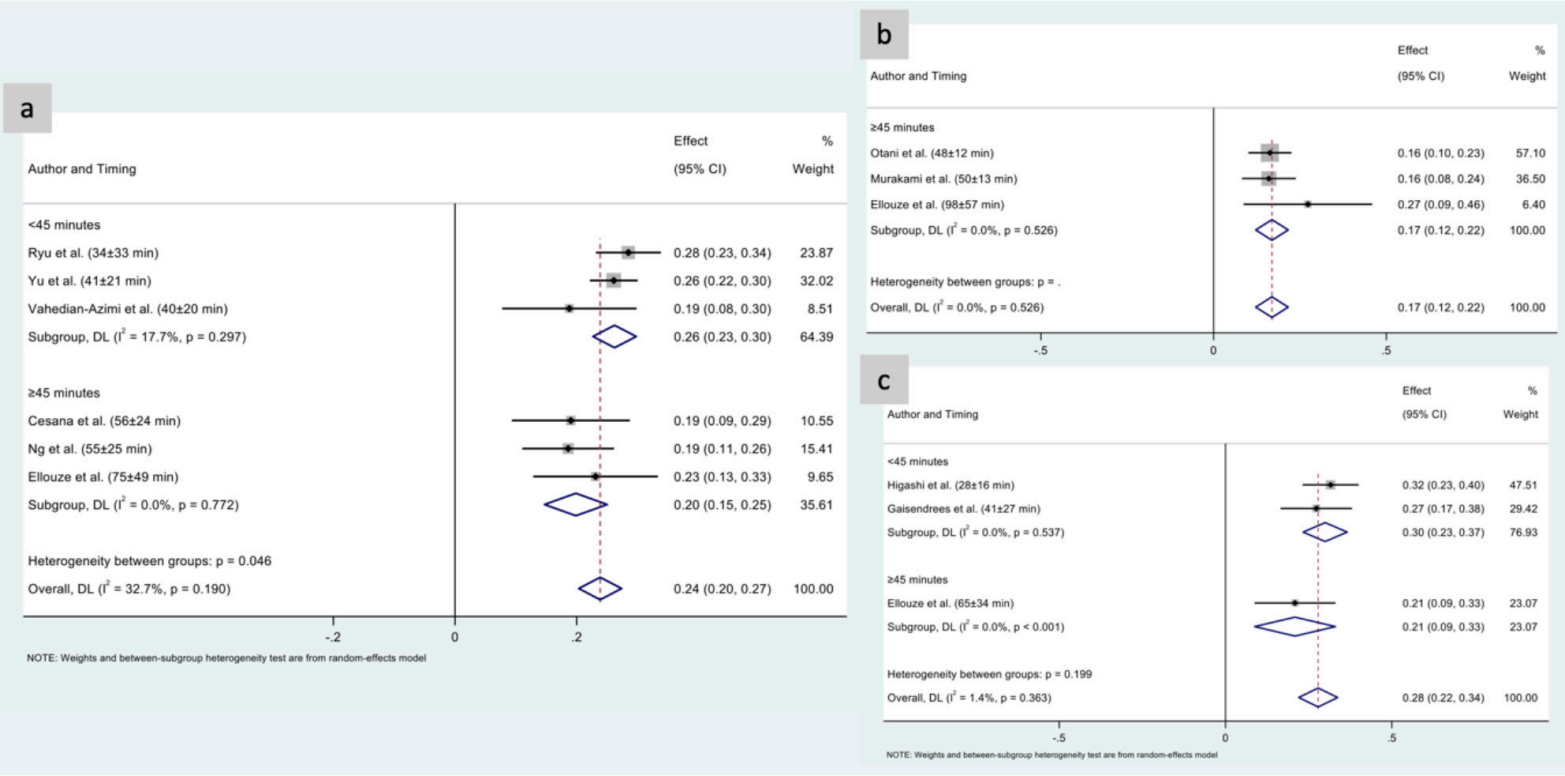
Forrest Plot of correlation between LFD with Neurological Outcomes in a) Both Groups; b) OHCA only patients, and c) IHCA only patients

Patients with LFD <45 minutes had higher survival rates than those with longer durations (p = 0.034; pooled I^2^ = 79.7% overall; I^2^ = 51.6% in the <45-min group and 67.7% in the ≥45-min group). This pattern held across both arrest locations: OHCA-only patients showed reduced survival with prolonged LFD (pooled effect 0.20; 95% CI: 0.11–0.29; I^2^ = 76.1%), and IHCA patients showed similarly poor survival when LFD exceeded 45 minutes (pooled effect 0.27; 95% CI: 0.14–0.40; I^2^ = 79.9%) [Figure 5]. These findings are consistent with an association between shorter LFD and more favorable survival, although the observational study designs preclude causal inference (see Discussion). Hospital LOS for the LFD analysis showed a pooled mean of 12.39 days (95% CI: 4.54–20.23; I^2^ = 82.3%) [Figure 6a].

**Figure 5.**
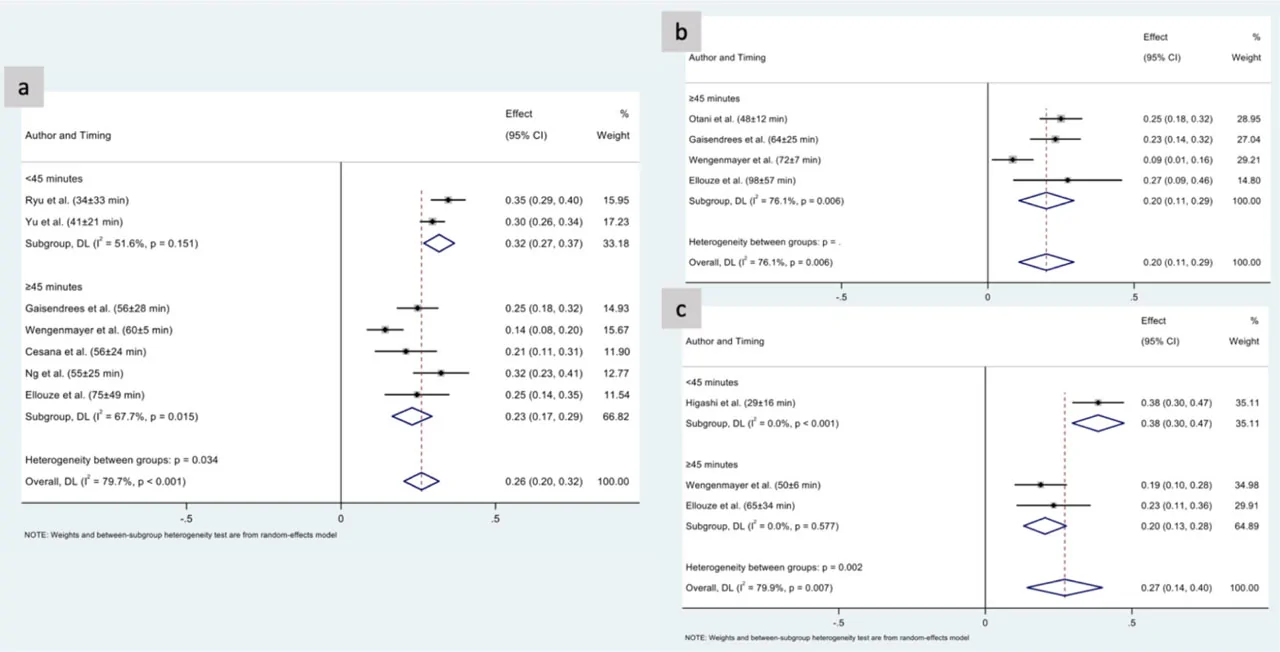
Forrest Plot of correlation between LFD with Survival at Discharge in a) Both Groups; b) OHCA only patients, and c) IHCA only patients

**Figure 6.**
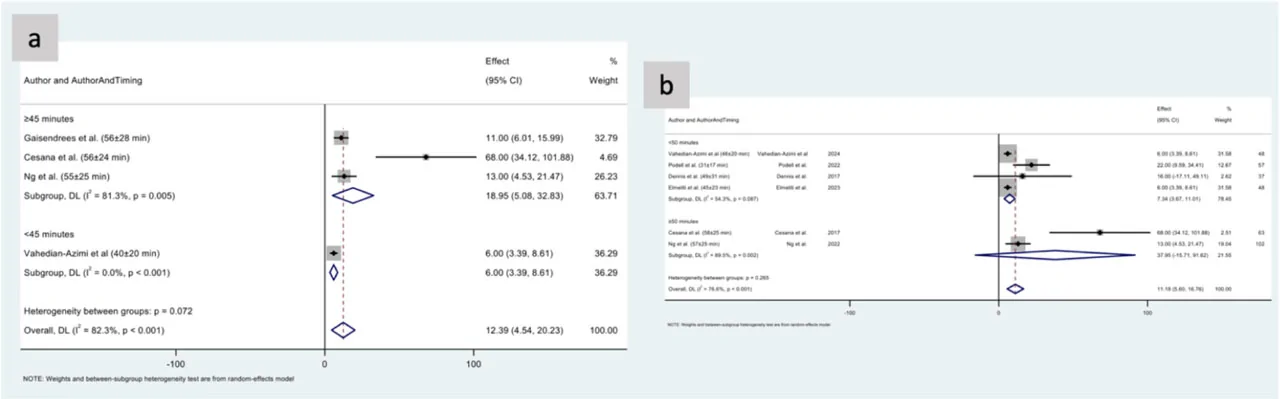
Forrest Plot of correlation between a) LFD; b) CA-to-ECMO with Hospital Length of Stay in Both Groups

### Cardiac arrest to ECMO duration

Meta-regression of CA-to-ECMO as a continuous predictor showed non-significant inverse associations with neurological outcomes (β = −0.571; p = 0.291; R^2^ = 0.218) and survival at discharge (β = −0.468; p = 0.398; R^2^ = 0.183). The meta-analysis, however, demonstrated that prolonged duration was associated with reduced survival (pooled effect = 0.33; 95% CI: 0.24–0.43) [Supplementary Figure 1d]. Subgroup comparisons of <50 versus ≥50 minutes revealed no significant difference (p ≈ 0.97–0.99), though favorable outcomes were observed more frequently with earlier cannulation (28% for <50 minutes) [Supplementary Figure 1b], with the highest survival reported by Podell et al. (45.6% at 31 minutes). In OHCA-only patients, subgroup analysis confirmed a significant association with survival at discharge (pooled effect = 0.29; 95% CI: 0.21–0.36; p < 0.001), with low heterogeneity (I^2^ = 25.1%, p = 0.246) [Supplementary Figure 2d]. Prolonged resuscitation was also associated with extended hospital stays, as patients with CA-to-ECMO <50 minutes had significantly shorter LOS (7.34 days; 95% CI: 3.67–11.01) compared with those ≥50 minutes (37.95 days; 95% CI: −15.71 to 91.62; this wide CI reflects the very small number of contributing studies), although meta-regression found no significant linear association (p = 0.519) [Figure 6b].

### Sensitivity Analyses and Publication Bias

Pooled estimates for all primary outcomes remained stable across leave-one-out iterations. For BOTH-group analyses, the largest single-study influence changed point estimates by 0.015–0.029 (pooled proportion scale) without altering the direction or statistical significance of any effect. Full leave-one-out results, including stratum-specific ranges, are presented in Supplementary Table S5 and Supplementary Figure S6.

Egger’s regression test was formally interpretable for only one outcome (OHCA CA-to-ECMO × favorable neurological outcome; k = 10), yielding an intercept p = 0.414, indicating no statistical evidence of small-study asymmetry. For all other outcomes (k < 10), Egger’s test was performed as an exploratory statistic only and should not be interpreted as confirmatory evidence for or against publication bias. Funnel plots for all outcomes with k ≥3 are presented in Supplementary Figure S5; no marked asymmetry was detected visually. For NFD-related analyses (k ≤8), formal publication-bias testing was not performed, and publication bias cannot be excluded.

### Certainty of Evidence (GRADE)

The certainty of evidence for each primary timing and outcome association is summarized in Supplementary Table S2. Across all eight combinations evaluated (NFD, LFD, and CA-to-ECMO versus favorable neurological outcome and survival at discharge, plus LFD and CA-to-ECMO versus hospital LOS), certainty was rated as LOW (⊕⊕○○) for most outcomes, driven primarily by the serious risk-of-bias downgrade applied to all observational studies. NFD-related outcomes and hospital LOS analyses received additional imprecision downgrades due to wide confidence intervals and small contributing sample sizes. Publication-bias domains were not downgraded because no statistical evidence of asymmetry was detected in the one outcome eligible for formal Egger testing, and funnel plots were visually symmetric for other outcomes.

## Discussion

In this systematic review and meta-analysis of 23 studies (n = 3,196) of patients receiving ECPR for refractory cardiac arrest, we examined the associations between three physiologically distinct timing parameters — NFD, LFD, and CA-to-ECMO — and survival at discharge, favorable neurological outcome, and hospital length of stay. LFD showed the most consistent association with outcomes; NFD and CA-to-ECMO showed directionally consistent but non-significant associations. We discuss each parameter separately below, given their distinct physiological and system-level determinants, and then consider the clinical, methodological, and economic implications of the pooled findings.

LFD showed the most consistent association with outcomes of the three parameters examined. In dichotomized analyses, LFD <45 minutes was associated with higher survival (32% vs. 23%; p = 0.034) and improved neurological recovery (26% vs. 20%; p = 0.046) across both IHCA and OHCA populations. Meta-regression demonstrated an inverse association between LFD and survival (β = −0.564; p = 0.024; R^2^ = 0.673), with progressively less favorable outcomes across increasing LFD durations, particularly in studies reporting LFDs beyond approximately 45–55 minutes, aligning with previous findings [8,15,16]. The underlying pathophysiology of prolonged ischemia is consistent with this association [3,24]. Given that conventional CPR provides only limited perfusion—typically 20–30% of normal cardiac output, insufficient to maintain vital organ perfusion organs [3,25], defibrillation becomes increasingly ineffective beyond 10 minutes, supporting the observed association between prolonged LFD and poorer outcomes [26,27].

Two caveats are important when interpreting the LFD finding. First, in observational data, shorter LFD is unlikely to be an isolated determinant of outcome; rather, it functions in part as a surrogate marker for other favorable patient and system characteristics, including initial shockable rhythm, witnessed arrest, rapid EMS response, short time to first defibrillation, and higher-performing ECPR systems. Patients who achieve shorter LFD are systematically different from those who do not, and a portion of the observed outcome difference therefore reflects confounding by indication and selection rather than the timing interval per se. Second, the absence of individual-patient data across the included studies prevents us from adjusting for these factors at the patient level. Consequently, our findings establish that shorter LFD is associated with more favorable outcomes but do not establish that reducing LFD in isolation would produce a proportional benefit, and must be interpreted as hypothesis-generating rather than causal. Furthermore, because the meta-regression used aggregate study-level data rather than individual patient-level observations, the observed associations should not be interpreted as patient-level dose-response relationships. Residual ecological bias (ecological fallacy) remains possible despite subgroup and sensitivity analyses.

No statistically significant association was observed between No-flow duration (NFD) and clinical outcomes in the present analysis. However, only a small number of studies contributed comparable NFD data, substantially limiting statistical power and robustness. Although prior studies have suggested worse outcomes with prolonged NFD [28, 29, 30], the available evidence in the present meta-analysis remains insufficient to draw firm conclusions. Accordingly, these findings should be considered exploratory and hypothesis-generating rather than confirmatory.

CA-to-ECMO time, encompassing both no flow and low flow phases, further modulates prognosis. Although not statistically significant in our analysis, low-heterogeneity subgroups revealed favorable trends. Yukawa et al. [31] found improved survival when CA-to-ECMO time was ≤33 minutes, and Zakhary et al. [32] noted significantly shorter times among survivors. Individual studies and guideline documents have proposed shorter CA-to-ECMO targets, including ≤33 minutes [31] and <40 minutes [17]. However, these thresholds were not directly validated in our pooled analysis and should be interpreted as observationally derived targets rather than definitive practice cutoffs. In our dataset, shorter CA-to-ECMO durations were generally associated with more favorable outcomes, although substantial heterogeneity limits firm threshold-based interpretation.

Identification and treatment of reversible causes remains important in ECPR. Several studies report that coronary angiography and/or PCI, whether before or after cannulation [31,33, 34, 35, 36, 37], is associated with better outcomes in selected patients with suspected coronary occlusion, and current guidelines recommend CAG chiefly for shockable rhythms, cardiogenic shock, or STEMI after ROSC [38, 39, 40, 41]. Consistent with this, favourable neurological outcomes have been reported even after prolonged LFD when revascularisation was performed [20]. Given the observational and descriptive basis of these data, however, they are better regarded as supporting prospective evaluation of CAG/PCI within ECPR pathways than as protocol-specific recommendations arising from our analysis.

Our subgroup analyses for both OHCA and IHCA populations demonstrated that LFD had the strongest and most consistent association with outcomes. For OHCA patients, studies reporting longer LFD duration, particularly beyond approximately 70 minutes, generally observed lower survival rates. For IHCA patients, less favorable outcomes were generally observed in studies reporting longer LFD durations, including those beyond approximately 45 minutes, with nearly double the survival rate observed with LFD of 29 minutes versus 50 minutes (38.8% vs. 18.9%) [42,43]. These findings highlight how arrest location influences both timing and prognosis [Figure 7]. IHCA cases benefit from rapid intervention but often involve comorbid patients [44]. In contrast, OHCA cases suffer delays in recognition and cannulation, though arrest location alone is not independently predictive of outcomes [43,45]. Thus, while timing remains essential, it is not sufficient on its own. Outcomes are also shaped by age, arrest etiology, comorbidities, and presenting rhythm [23,46, 47, 48].

**Figure 7.**
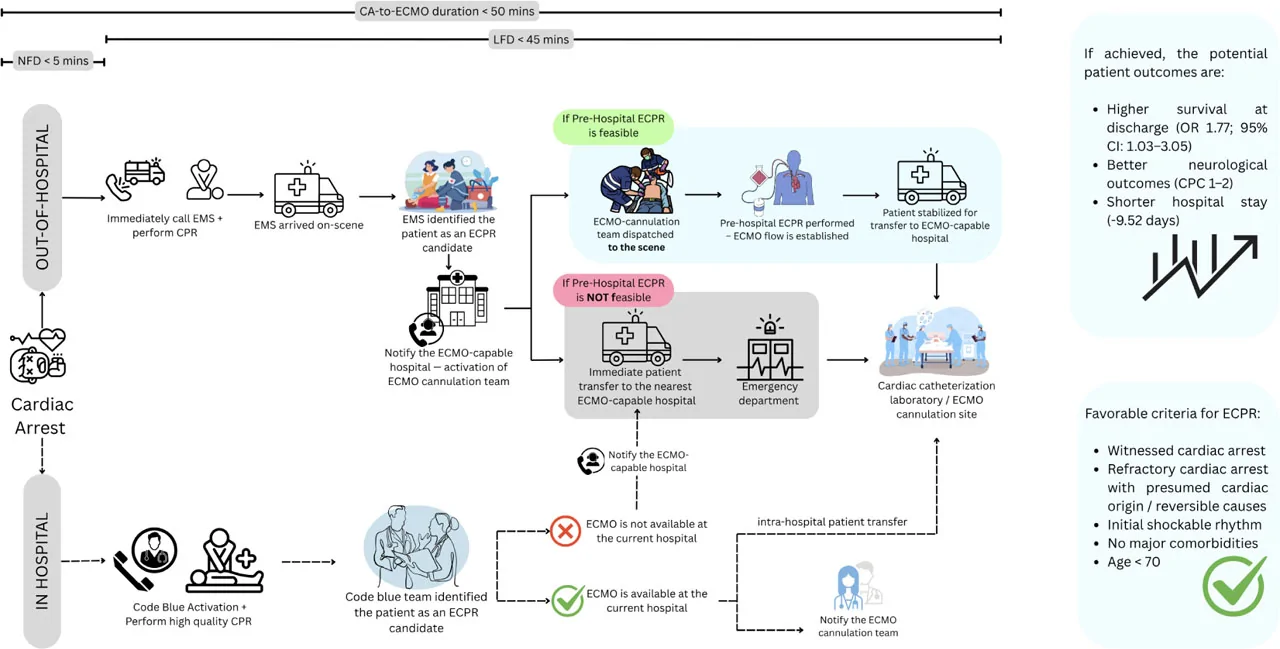
Illustration of ECPR program for in- and out-of-hospital cardiac arrest

ECMO is a resource-intensive, technically complex intervention requiring trained personnel and cannot be universally implemented; it is best performed in high-volume centers. ECPR demands immediate activation, coordinated teamwork, and predefined roles, as emphasized by American Heart Association (AHA) guidelines, which include a trained cannulation team and a supervising team leader to manage concurrent resuscitation efforts [17]. Accordingly, recent international guidelines have narrowed ECPR indications to optimize value. The European Resuscitation Council recommends ECPR in patients with presumed reversible causes, witnessed arrests, immediate CPR, and LFD <60 minutes [5].

These clinical priorities must be considered in light of mounting economic scrutiny. A cost-effectiveness analysis of the INCEPTION trial estimated an incremental cost of €48,891 per QALY gained with ECPR [49], median cost of AUD 61,294 per OHCA case and median hospitalization costs of USD 105,529 [50]. Prolonged hospitalization further compounds this economic burden, especially in patients with poor outcomes [51,52]. These findings underscore the need to optimize timing and selection criteria in ECPR to enhance both clinical efficacy and cost-effectiveness.

Pre-hospital ECPR has emerged as a promising strategy to minimize LFD in OHCA cases. This approach enables ECMO support within 60 minutes of arrest, with more favorable outcomes reported in programs achieving shorter LFD durations, including those under approximately 30 minutes [53, 54, 55]. Observational studies and feasibility trials—including the PRECARE study in Sydney, CHEER3 in Melbourne, and programs in Paris, the Netherlands, and Regensburg—have reported favorable neurological outcomes (CPC 1–2) in 25% to 40% of carefully selected patients [53,54,56]. These programs rely on specialized pre-hospital teams, typically comprising two physicians (anesthesiologist, intensivist, or emergency medicine specialist) for cannulation and a critical care paramedic for circuit management [54,56].

This growing consensus reflects a shared view that ECPR must be delivered quickly, selectively, and within an organized framework. Rodrigues et al.[57] highlighted that harmonizing time-based thresholds and institutional protocols is crucial for ensuring equitable and effective ECPR deployment. Decision-analytic modeling supports this: a Canadian study showed that ECPR becomes cost-effective only when favorable neurologic outcomes exceed 18%, reinforcing the importance of judicious patient selection [58]. Despite international recommendations, resuscitation practices vary across institutions based on the experience of the clinical team, resources, and the readiness of ECLS systems [59].

This meta-analysis has several important limitations. First, substantial clinical heterogeneity existed across included studies, including differences in cardiac arrest setting, activation systems, inclusion criteria, witness status, initial rhythm, CPR quality, cannulation techniques, and post-ECPR management, which could not be fully addressed by IHCA/OHCA stratification alone and likely contributed to wide confidence intervals. Second, most evidence was derived from observational studies, making shorter timing intervals susceptible to confounding by patient- and system-level factors, particularly the surrogate role of LFD. Because the meta-regression was performed on study-level aggregate data rather than individual-patient data, the observed associations are ecological and may not hold at the patient level; they should not be interpreted as patient-level dose–response relationships, and residual ecological bias cannot be excluded despite the precautions taken. Third, the NFD analysis included only eight studies, limiting statistical power and representativeness. In addition, most studies were retrospective, single-centre, and conducted in high-volume specialized institutions, reducing generalizability. Various timing definitions across studies may also have introduced residual misclassification. Publication bias could not be formally assessed for outcomes with fewer than 10 studies. Overall, GRADE certainty ranged from low to very low, so these findings should be considered hypothesis-generating and require prospective multi-centre validation.

Future research priorities include standardization of timing parameter definitions, prospective validating identified thresholds through multi-center registries, and exploration of CPR quality’s role during low-flow periods Comprehensive predictive models integrating timing variables with patient characteristics are needed, alongside comparative studies of deployment strategies, particularly pre-hospital ECPR, and economic evaluations using refined thresholds to inform optimal, sustainable program expansion across diverse health-care systems.

## Conclusion

Across 23 studies included in this systematic review and meta-analysis, shorter LFD was consistently associated with improved survival and favorable neurological outcomes following ECPR. Outcomes generally declined with increasing LFD, although these findings should be interpreted cautiously due to substantial between-study heterogeneity, differences in ECPR systems, patient selection, cannulation practices, and arrest context. While shorter CA-to-ECMO durations appeared to be associated with more favorable outcomes, the available evidence remains insufficient to support a definitive universal time threshold. In contrast, the evidence regarding NFD was limited by the small number of studies reporting comparable data. Overall, these findings support further prospective evaluation of strategies aimed at reducing low-flow duration and improving ECMO deployment systems rather than establishing definitive clinical timing targets.

## Supplementary Material

Supplementary Material Details

## References

[1] Tsao CW, Aday AW, Almarzooq ZI, Alonso A, Beaton AZ, Bittencourt MS (2022). Heart Disease and Stroke Statistics-2022 Update: A Report from the American Heart Association. Circulation.

[2] Penketh J, Nolan JP (2022). In-hospital cardiac arrest: the state of the art. Crit Care.

[3] Jiang L, Zhang J (2011). Mechanical cardiopulmonary resuscitation for patients with cardiac arrest. World J Emerg Med.

[4] Richardson ASC, Tonna JE, Nanjayya V, Nixon P, Abrams DC, Raman L (2021). Extracorporeal Cardiopulmonary Resuscitation in Adults. Interim Guideline Consensus Statement From the Extracorporeal Life Support Organization. ASAIO J.

[5] Soar J, Maconochie I, Wyckoff MH, Olasveengen TM, Singletary EM, Greif R (2019). 2019 International Consensus on Cardiopulmonary Resuscitation and Emergency Cardiovascular Care Science With Treatment Recommendations. Resuscitation.

[6] Kim SJ, Kim HJ, Lee HY, Ahn HS, Lee SW (2016). Comparing extracorporeal cardiopulmonary resuscitation with conventional cardiopulmonary resuscitation: A meta-analysis. Resuscitation.

[7] Sakamoto T, Morimura N, Nagao K, Asai Y, Yokota H, Nara S (2014). Extracorporeal cardiopulmonary resuscitation versus conventional cardiopulmonary resuscitation in adults with out-of-hospital cardiac arrest: A prospective observational study. Resuscitation.

[8] Shoji K, Ohbe H, Matsuyama T, Inoue A, Hifumi T, Sakamoto T (2024). Low-flow time and outcomes in hypothermic cardiac arrest patients treated with extracorporeal cardiopulmonary resuscitation: a secondary analysis of a multi-center retrospective cohort study. J Intensive Care.

[9] Yannopoulos D, Bartos J, Raveendran G, Walser E, Connett J, Murray TA (2020). Advanced reperfusion strategies for patients with out-of-hospital cardiac arrest and refractory ventricular fibrillation (ARREST): a phase 2, single centre, open-label, randomised controlled trial. The Lancet.

[10] Scquizzato T, Bonaccorso A, Consonni M, Scandroglio AM, Swol J, Landoni G (2022). Extracorporeal cardiopulmonary resuscitation for out-of-hospital cardiac arrest: A systematic review and meta-analysis of randomized and propensity score-matched studies. Artif Organs.

[11] Leick J, Liebetrau C, Szardien S, Fischer-Rasokat U, Willmer M, Van Linden A (2013). Door-to-implantation time of extracorporeal life support systems predicts mortality in patients with out-of-hospital cardiac arrest. Clinical Research in Cardiology.

[12] Ha TS, Yang JH, Cho YH, Chung CR, Park CM, Jeon K (2017). Clinical outcomes after rescue extracorporeal cardiopulmonary resuscitation for out-of-hospital cardiac arrest. Emergency Medicine Journal.

[13] Fagnoul D, Combes A, De Backer D (2014). Extracorporeal cardiopulmonary resuscitation. Curr Opin Crit Care.

[14] Komatsu T, Kinoshita K, Sakurai A, Moriya T, Yamaguchi J, Sugita A (2014). Shorter time until return of spontaneous circulation is the only independent factor for a good neurological outcome in patients with postcardiac arrest syndrome. Emergency Medicine Journal.

[15] Park IH, Yang JH, Jang WJ, Chun WJ, Oh JH, Park YH (2020). Clinical Significance of Low-Flow Time in Patients Undergoing Extracorporeal Cardiopulmonary Resuscitation: Results from the RESCUE Registry. J Clin Med.

[16] Ohbe H, Tagami T, Ogura T, Matsui H, Yasunaga H (2022). Low-Flow Duration and Outcomes of Extracorporeal Cardiopulmonary Resuscitation in Adults With In-Hospital Cardiac Arrest: A Nationwide Inpatient Database Study?. Crit Care Med.

[17] Perman SM, Elmer J, Maciel CB, Uzendu A, May T, Mumma BE (2024). 2023 American Heart Association Focused Update on Adult Advanced Cardiovascular Life Support: An Update to the American Heart Association Guidelines for Cardiopulmonary Resuscitation and Emergency Cardiovascular Care. Circulation.

[18] Sterne JAC, Savović J, Page MJ, Elbers RG, Blencowe NS, Boutron I (2019). RoB 2: A revised tool for assessing risk of bias in randomised trials. The BMJ.

[19] Sterne JA, Hernán MA, Reeves BC, Savović J, Berkman ND, Viswanathan M (2016). ROBINS-I: A tool for assessing risk of bias in non-randomised studies of interventions. BMJ (Online).

[20] Fjølner J, Greisen J, Jørgensen MRS, Terkelsen CJ, Ilkjær LB, Hansen TM (2017). Extracorporeal cardiopulmonary resuscitation after out-of-hospital cardiac arrest in a Danish health region. Acta Anaesthesiol Scand.

[21] Podell JE, Krause EM, Rector R, Hassan M, Reddi A, Jaffa MN (2022). Neurologic Outcomes after Extracorporeal Cardiopulmonary Resuscitation: Recent Experience at a Single High-Volume Center. ASAIO Journal.

[22] Rob D, Smalcova J, Smid O, Kral A, Kovarnik T, Zemanek D (2022). Extracorporeal versus conventional cardiopulmonary resuscitation for refractory out-of-hospital cardiac arrest: a secondary analysis of the Prague OHCA trial. Crit Care.

[23] Ubben JFH, Suverein MM, Delnoij TSR, Heuts S, Winkens B, Gabrio A (2024). Early extracorporeal CPR for refractory out-of-hospital cardiac arrest – A pre-planned per-protocol analysis of the INCEPTION-trial. Resuscitation.

[24] Welbourn C, Efstathiou N (2018). How does the length of cardiopulmonary resuscitation affect brain damage in patients surviving cardiac arrest? A systematic review. Scand J Trauma Resusc Emerg Med.

[25] Meaney PA, Bobrow BJ, Mancini ME, Christenson J, De Caen AR, Bhanji F (2013). Cardiopulmonary resuscitation quality: Improving cardiac resuscitation outcomes both inside and outside the hospital: A consensus statement from the American heart association. Circulation.

[26] Wallentin L, Kristensen SD, Anderson JL, Tubaro M, Sendon JLL, Granger CB (2014). How can we optimize the processes of care for acute coronary syndromes to improve outcomes?. Am Heart J.

[27] Patil KD, Halperin HR, Becker LB (2015). Cardiac Arrest: Resuscitation and Reperfusion. Circ Res.

[28] Kawakami S, Tahara Y, Koga H, Noguchi T, Inoue S, Yasuda S (2022). The association between time to extracorporeal cardiopulmonary resuscitation and outcome in patients with out-of-hospital cardiac arrest. Eur Heart J Acute Cardiovasc Care.

[29] Assouline B, Mentha N, Wozniak H, Donner V, Looyens C, Suppan L (2024). Improved Extracorporeal Cardiopulmonary Resuscitation (ECPR) Outcomes are Associated with a Restrictive Patient Selection Algorithm. J Clin Med.

[30] Perkins GD, Callaway CW, Haywood K, Neumar RW, Lilja G, Rowland MJ (2021). Brain injury after cardiac arrest. The Lancet.

[31] Yukawa T, Kashiura M, Sugiyama K, Tanabe T, Hamabe Y (2017). Neurological outcomes and duration from cardiac arrest to the initiation of extracorporeal membrane oxygenation in patients with out-of-hospital cardiac arrest: A retrospective study. Scand J Trauma Resusc Emerg Med.

[32] Zakhary B, Nanjayya VB, Sheldrake J, Collins K, Ihle JF, Pellegrino V (2018). Predictors of mortality after extracorporeal cardiopulmonary resuscitation.

[33] Murakami N, Kokubu N, Nagano N, Nishida J, Nishikawa R, Nakata J (2020). Prognostic Impact of No-Flow Time on 30-Day Neurological Outcomes in Patients With Out-of-Hospital Cardiac Arrest Who Received Extracorporeal Cardiopulmonary Resuscitation. Circulation Journal.

[34] Otani T, Sawano H, Natsukawa T, Nakashima T, Oku H, Gon C (2018). Low-flow time is associated with a favorable neurological outcome in out-of-hospital cardiac arrest patients resuscitated with extracorporeal cardiopulmonary resuscitation. J Crit Care.

[35] Shih HM, Lin WJ, Lin YC, Chang SS, Chang KC, Yu SH (2024). Extracorporeal cardiopulmonary resuscitation for patients with refractory out-of-hospital cardiac arrest: a propensity score matching, observational study. Sci Rep.

[36] Siao FY, Chiu CC, Chiu CW, Chen YC, Chen YL, Hsieh YK (2015). Managing cardiac arrest with refractory ventricular fibrillation in the emergency department: Conventional cardiopulmonary resuscitation versus extracorporeal cardiopulmonary resuscitation. Resuscitation.

[37] Takahagi M, Sawano H, Moriyama T (2022). Long-Term Neurological Outcome of Extracorporeal Cardiopulmonary Resuscitation for Out-of-Hospital Cardiac Arrest Patients With Nonshockable Rhythms: A Single-Center, Consecutive, Retrospective Observational Study. Journal of Emergency Medicine.

[38] Lawton JS, Tamis-Holland JE, Bangalore S, Bates ER, Beckie TM, Bischoff JM (2022). 2021 ACC/AHA/SCAI Guideline for Coronary Artery Revascularization: A Report of the American College of Cardiology/American Heart Association Joint Committee on Clinical Practice Guidelines. J Am Coll Cardiol.

[39] Zeppenfeld K, Tfelt-Hansen J, De Riva M, Winkel BG, Behr ER, Blom NA (2022). 2022 ESC Guidelines for the management of patients with ventricular arrhythmias and the prevention of sudden cardiac death. Eur Heart J.

[40] Collet JP, Thiele H, Barbato E, Bauersachs J, Dendale P, Edvardsen T (2021). 2020 ESC Guidelines for the management of acute coronary syndromes in patients presenting without persistent ST-segment elevation. Eur Heart J.

[41] Nolan JP, Sandroni C, Böttiger BW, Cariou A, Cronberg T, Friberg H (2021). European Resuscitation Council and European Society of Intensive Care Medicine Guidelines 2021: Post-resuscitation care. Resuscitation.

[42] Higashi A, Nakada TA, Imaeda T, Abe R, Shinozaki K, Oda S (2020). Shortening of low-flow duration over time was associated with improved outcomes of extracorporeal cardiopulmonary resuscitation in in-hospital cardiac arrest. J Intensive Care.

[43] Wengenmayer T, Rombach S, Ramshorn F, Biever P, Bode C, Duerschmied D (2017). Influence of low-flow time on survival after extracorporeal cardiopulmonary resuscitation (eCPR). Crit Care.

[44] Carnicelli AP, Keane R, Brown KM, Loriaux DB, Kendsersky P, Alviar CL (2022). Characteristics, Therapies, and Outcomes of In-Hospital vs Out-of-Hospital Cardiac Arrest in Patients Presenting to Cardiac Intensive Care Units: From the Critical Care Cardiology Trials Network (CCCTN). Resuscitation.

[45] Andersson A, Arctaedius I, Cronberg T, Levin H, Nielsen N, Friberg H (2022). In-hospital versus out-of-hospital cardiac arrest: Characteristics and outcomes in patients admitted to intensive care after return of spontaneous circulation. Resuscitation.

[46] Cesana F, Avalli L, Garatti L, Coppo A, Righetti S, Calchera I (2018). Effects of extracorporeal cardiopulmonary resuscitation on neurological and cardiac outcome after ischaemic refractory cardiac arrest. Eur Heart J Acute Cardiovasc Care.

[47] Dennis M, McCanny P, D’Souza M, Forrest P, Burns B, Lowe DA (2017). Extracorporeal cardiopulmonary resuscitation for refractory cardiac arrest: A multicentre experience. Int J Cardiol.

[48] Choi Y, Park JH, Jeong J, Kim YJ, Song KJ, Shin S Do (2023). Extracorporeal cardiopulmonary resuscitation for adult out-of-hospital cardiac arrest patients: time-dependent propensity score-sequential matching analysis from a nationwide population-based registry. Crit Care.

[49] Delnoij TSR, Suverein MM, Essers BAB, Hermanides RC, Otterspoor L, Elzo Kraemer C V. (2024). Cost-effectiveness of extracorporeal cardiopulmonary resuscitation vs. conventional cardiopulmonary resuscitation in out-of-hospital cardiac arrest: A pre-planned, trial-based economic evaluation. Eur Heart J Acute Cardiovasc Care.

[50] Thevathasan T, Paul J, Gaul AL, Degbeon S, Füreder L, Dischl D (2023). Mortality and healthcare resource utilisation after cardiac arrest in the United States – A 10-year nationwide analysis prior to the COVID-19 pandemic. Resuscitation.

[51] Semedi BP, Ramadhania NN, Tambunan BA, Bintoro SUY, Soedarsono S, Prakoeswa CRS (2022). Prolonged ICU Stay in Severe and Critically-Ill COVID-19 Patients Who Received Convalescent Plasma Therapy. Crit Care Res Pract.

[52] Richardson SAC, Anderson D, Burrell AJC, Byrne T, Coull J, Diehl A (2023). Pre-hospital ECPR in an Australian metropolitan setting: a single-arm feasibility assessment—The CPR, pre-hospital ECPR and early reperfusion (CHEER3) study. Scand J Trauma Resusc Emerg Med.

[53] Kruit N, Burns B, Shearer N, Hui J, Coggins A, Buscher H (2025). Pre-hospital ECPR for refractory cardiac arrest – The PRECARE pilot feasibility study. Resuscitation.

[54] Jones D, Daglish FM, Tanner BM, Wilkie FJM (2024). A review of pre-hospital extracorporeal cardiopulmonary resuscitation and its potential application in the North East of England. Int J Emerg Med.

[55] Damluji AA, Al-Damluji MS, Pomenti S, Zhang TJ, Cohen MG, Mitrani RD (2018). Health Care Costs after Cardiac Arrest in the United States. Circ Arrhythm Electrophysiol.

[56] Rodrigues AR, Oliveira A, Vieira T, Assis R, Lume C, Gonçalves-Pereira J (2024). A prolonged intensive care unit stay defines a worse long-term prognosis – Insights from the critically ill mortality by age (Cimba) study. Australian Critical Care.

[57] Addison D, Cheng E, Forrest P, Livingstone A, Morton RL, Dennis M (2022). Cost-effectiveness of extracorporeal cardiopulmonary resuscitation for adult out-of-hospital cardiac arrest: A systematic review. Resuscitation.

[58] De Charrière A, Assouline B, Scheen M, Mentha N, Banfi C, Bendjelid K (2021). Ecmo in cardiac arrest: A narrative review of the literature. J Clin Med.

[59] Elmelliti H, Vahedian-Azimi A, Albazoon F, Alqahwachi H, Akbar A, Shehatta AL (2023). Outcomes of Patients With in- and out-of-hospital Cardiac Arrest on Extracorporeal Cardiopulmonary Resuscitation: A Single-center Retrospective Cohort Study. Curr Probl Cardiol.

[60] Fu Y, Chu SE, Chang CJ, Huang CY, Sim SS, Chiang WC (2024). 236 Optimization of Low-Flow Time in Extracorporeal Cardiopulmonary Resuscitation for Out-of-Hospital Cardiac Arrest: A Decade-Long Analysis. Ann Emerg Med.

[61] Gaisendrees C, Schlachtenberger G, Müller L, Jaeger D, Djordjevic I, Krasivskyi I (2024). Extracorporeal cardiopulmonary resuscitation for in- and out-of-hospital cardiac arrest: The race against time. Resusc Plus.

[62] Ng PY, Li ACC, Fang S, Lin JCR, Ip A, Chan WM (2022). Predictors of Favorable Neurologic Outcomes in a Territory-First Extracorporeal Cardiopulmonary Resuscitation Program. ASAIO Journal.

[63] Ryu JA, Chung CR, Cho YH, Sung K, Jeon K, Suh GY (2019). Neurologic Outcomes in Patients Who Undergo Extracorporeal Cardiopulmonary Resuscitation. Annals of Thoracic Surgery.

[64] Stub D, Bernard S, Pellegrino V, Smith K, Walker T, Sheldrake J (2015). Refractory cardiac arrest treated with mechanical CPR, hypothermia, ECMO and early reperfusion (the CHEER trial). Resuscitation.

[65] Vahedian-Azimi A, Hassan IF, Rahimi-Bashar F, Elmelliti H, Salesi M, Alqahwachi H (2024). Prognostic effects of cardiopulmonary resuscitation (CPR) start time and the interval between CPR to extracorporeal cardiopulmonary resuscitation (ECPR) on patient outcomes under extracorporeal membrane oxygenation (ECMO): a single-center, retrospective observational study. BMC Emerg Med.

[66] Yu HY, Wang CH, Chi NH, Huang SC, Chou HW, Chou NK (2019). Effect of interplay between age and low-flow duration on neurologic outcomes of extracorporeal cardiopulmonary resuscitation. Intensive Care Med.

[67] Puolakka T, Salo A, Varpula M, Nurmi J, Skrifvars MB, Wilkman E (2023). Hospital-administered ECPR for out-of-hospital cardiac arrest: An observational cohort study. Emergency Medicine Journal.

[68] Ellouze O, Vuillet M, Perrot J, Grosjean S, Missaoui A, Aho S (2018). Comparable Outcome of Out-of-Hospital Cardiac Arrest and In-Hospital Cardiac Arrest Treated With Extracorporeal Life Support. Artif Organs.

