# Supplementary material for: Impact of timing parameters on outcomes following extracorporeal cardiopulmonary resuscitation: a systematic review and meta-analysis
Source: J Crit Care Med (Targu Mures). 2026 Oct 30;12(4):528–45. doi: 10.62838/jccm-2026-0049 (PMC13752159; doi:10.62838/jccm-2026-0049)

Supplementary Material

Supplementary Figure 1 – Forest Plots of OHCA & IHCA (Both) Groups

a) Forrest Plot of correlation between NFD with Neurological Outcomes

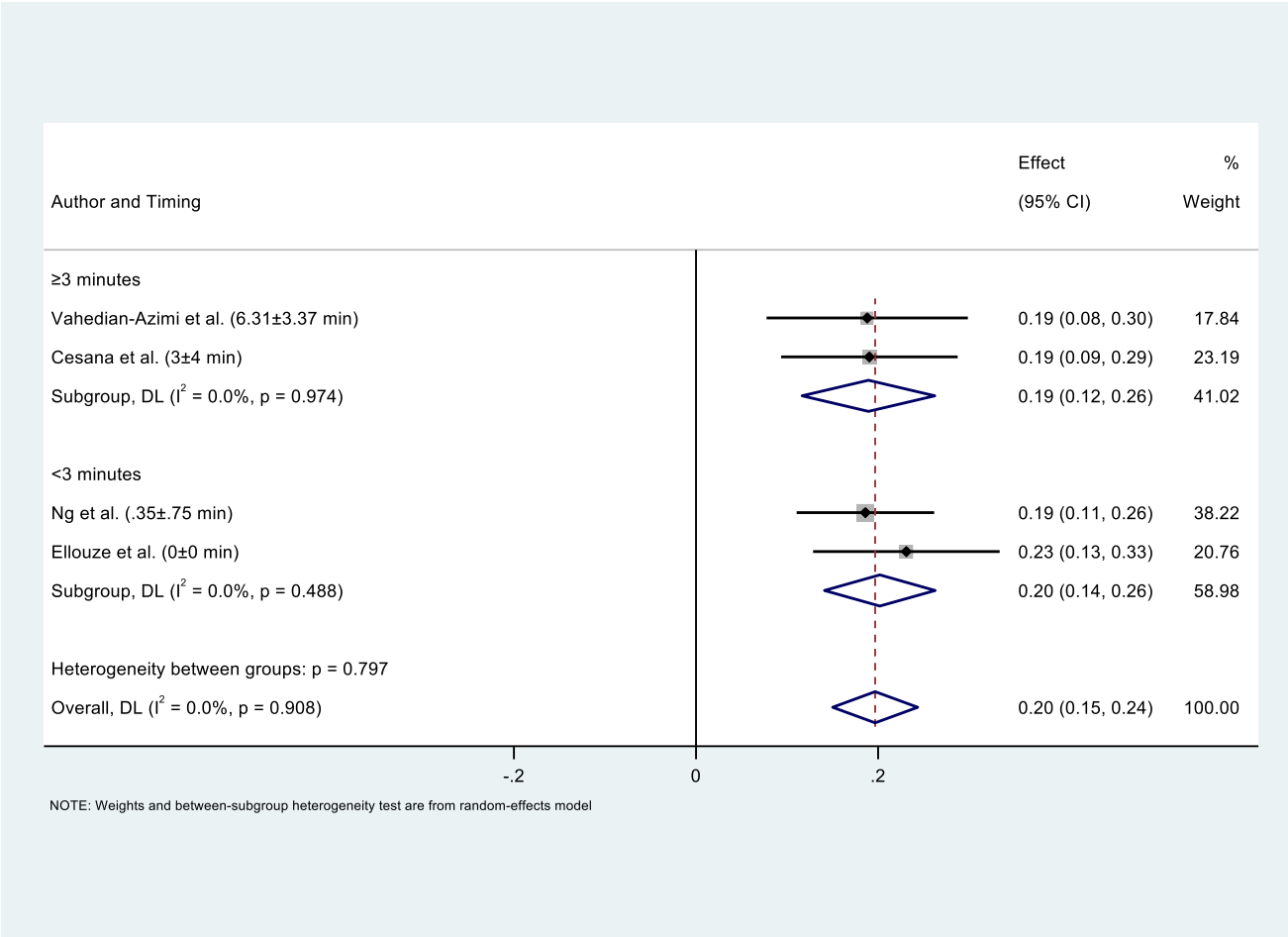

b) Forrest Plot of correlation between CA to ECMO Duration with Neurological Outcomes

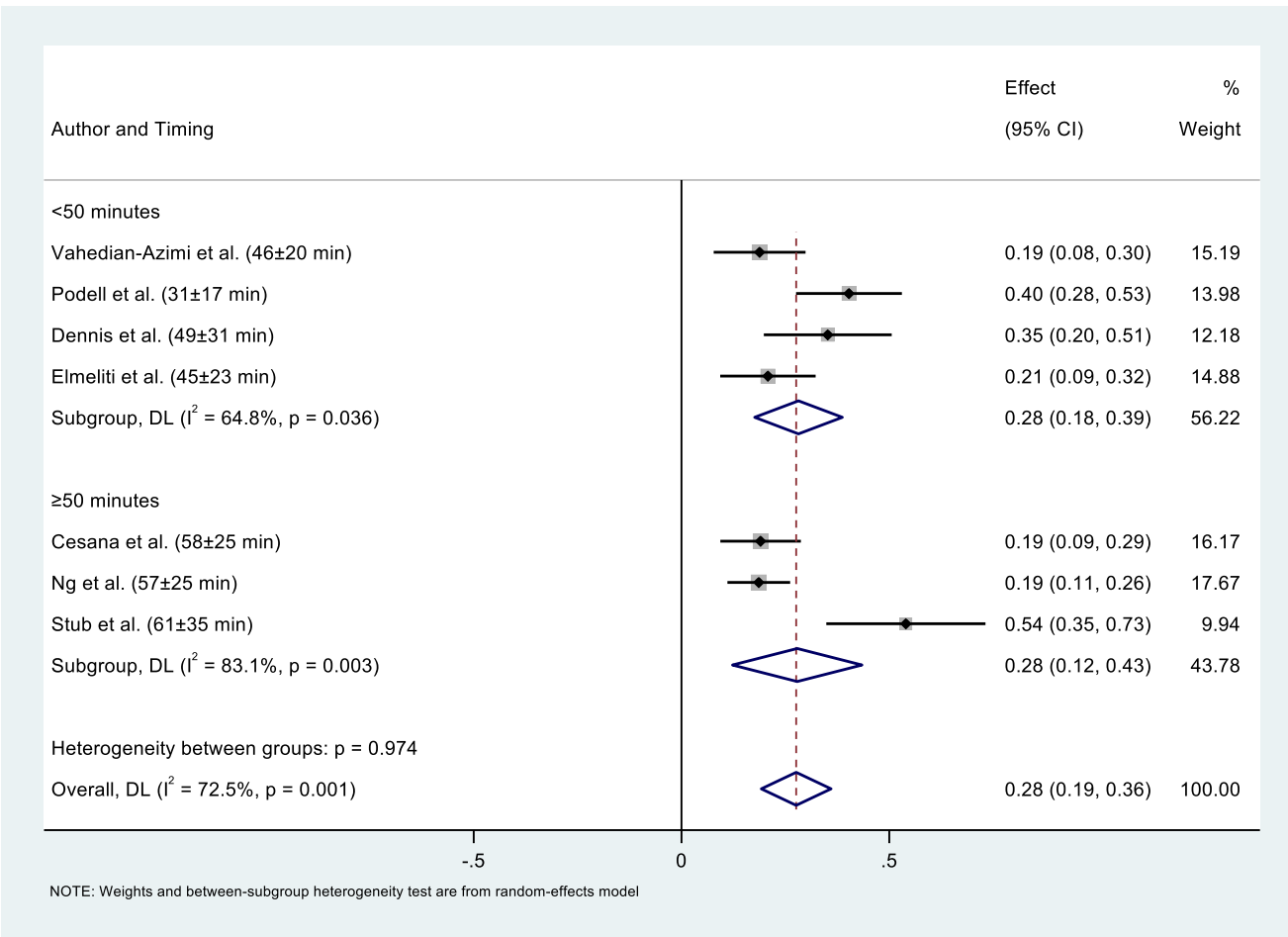

c) Forrest Plot of correlation between NFD with Survival at Discharge

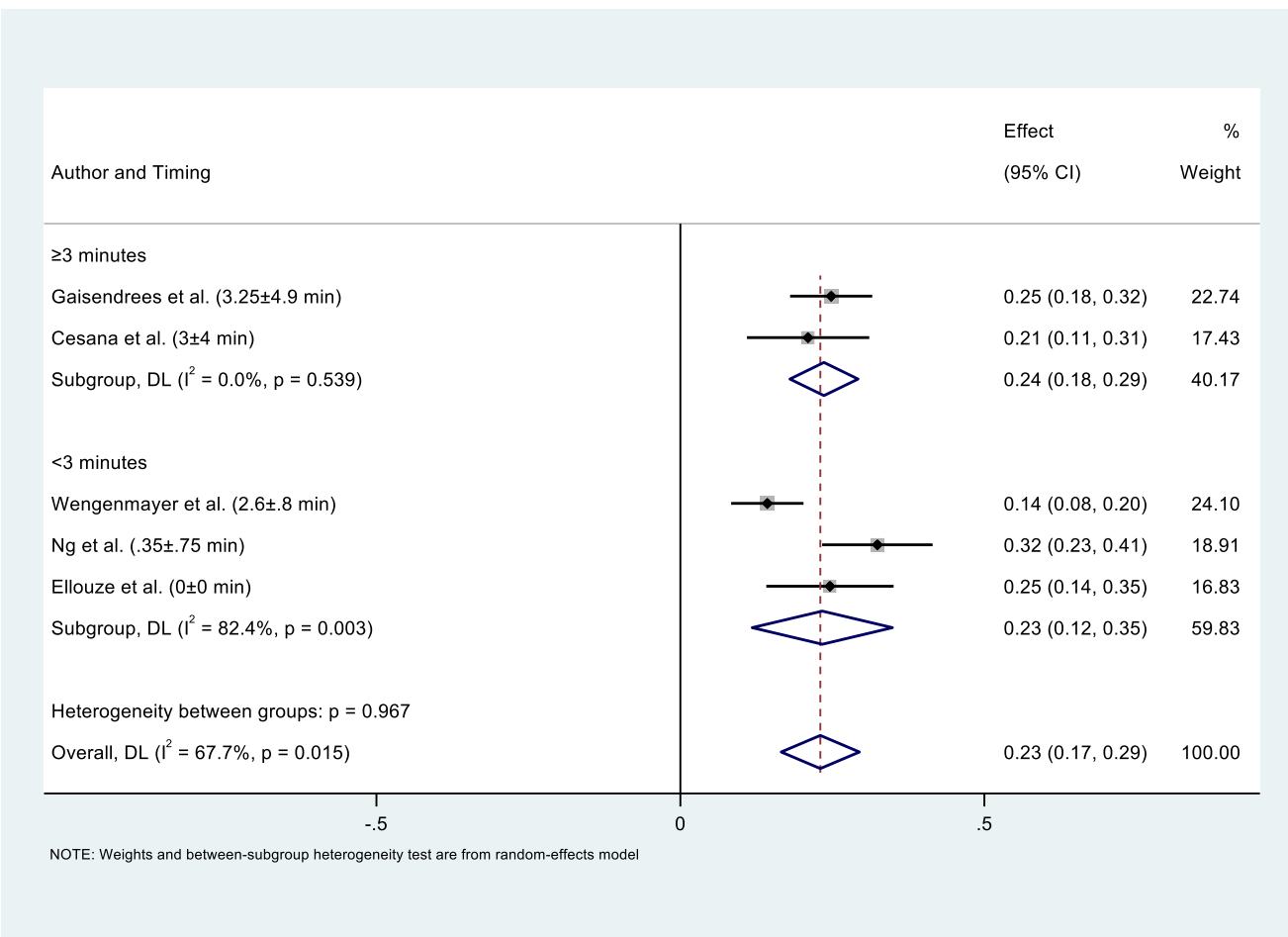

d) Forrest Plot of correlation between CA to ECMO Duration with Survival at Discharge

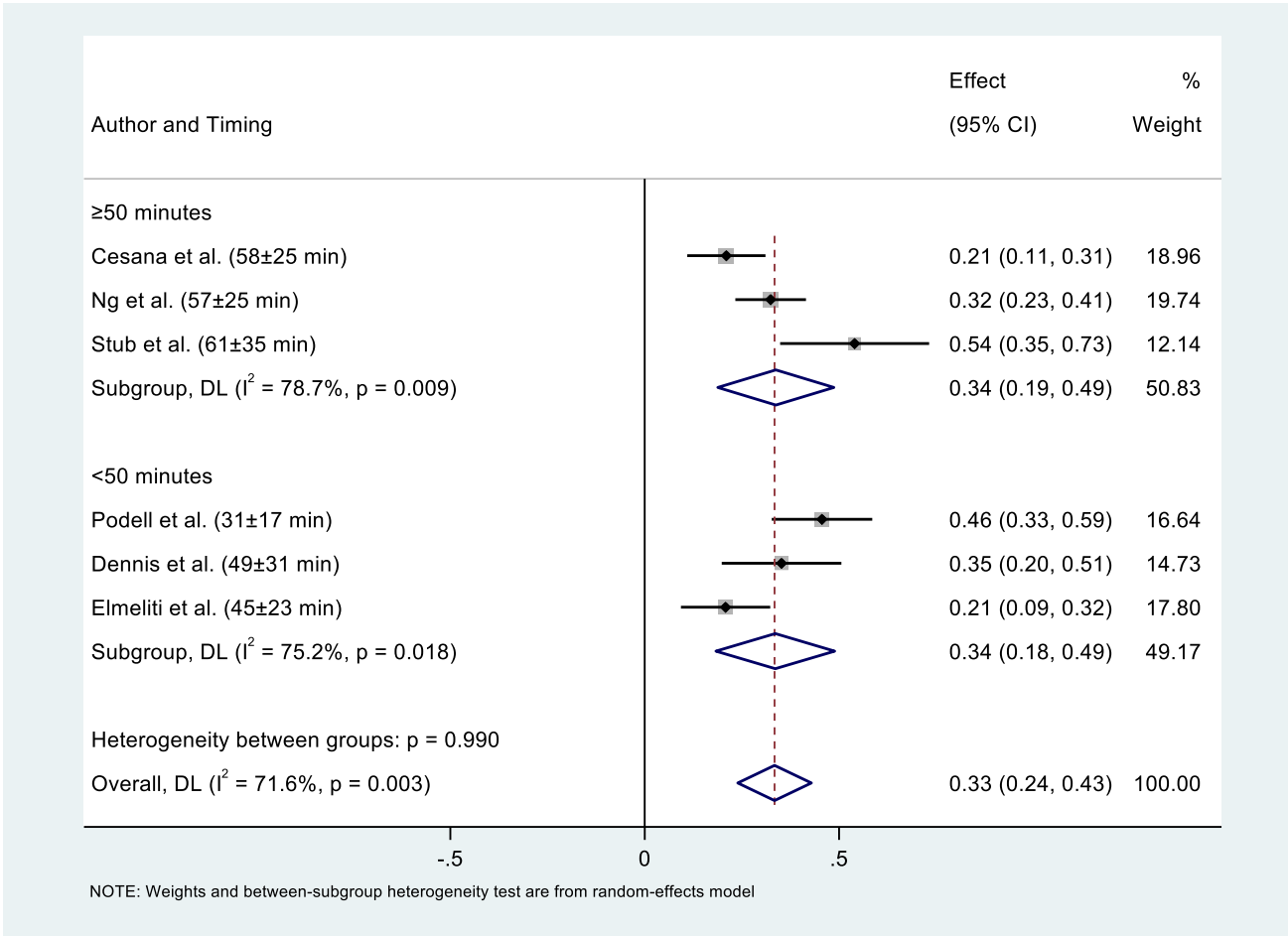

Supplementary Figure 2 – Forest Plots of OHCA Only Groups

a) Forrest Plot of correlation between NFD with Neurological Outcomes

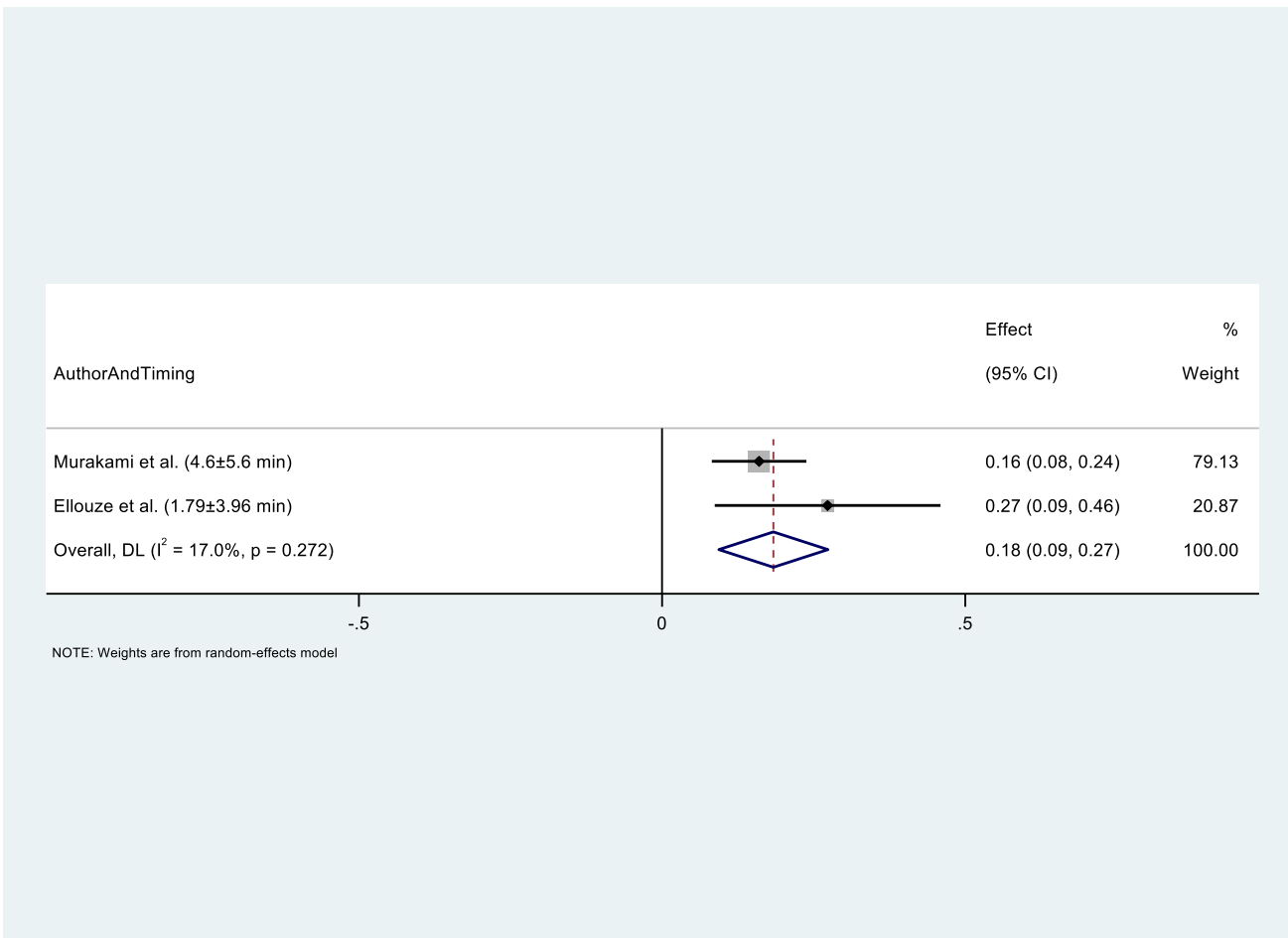

b) Forrest Plot of correlation between CA to ECMO Duration with Neurological Outcomes

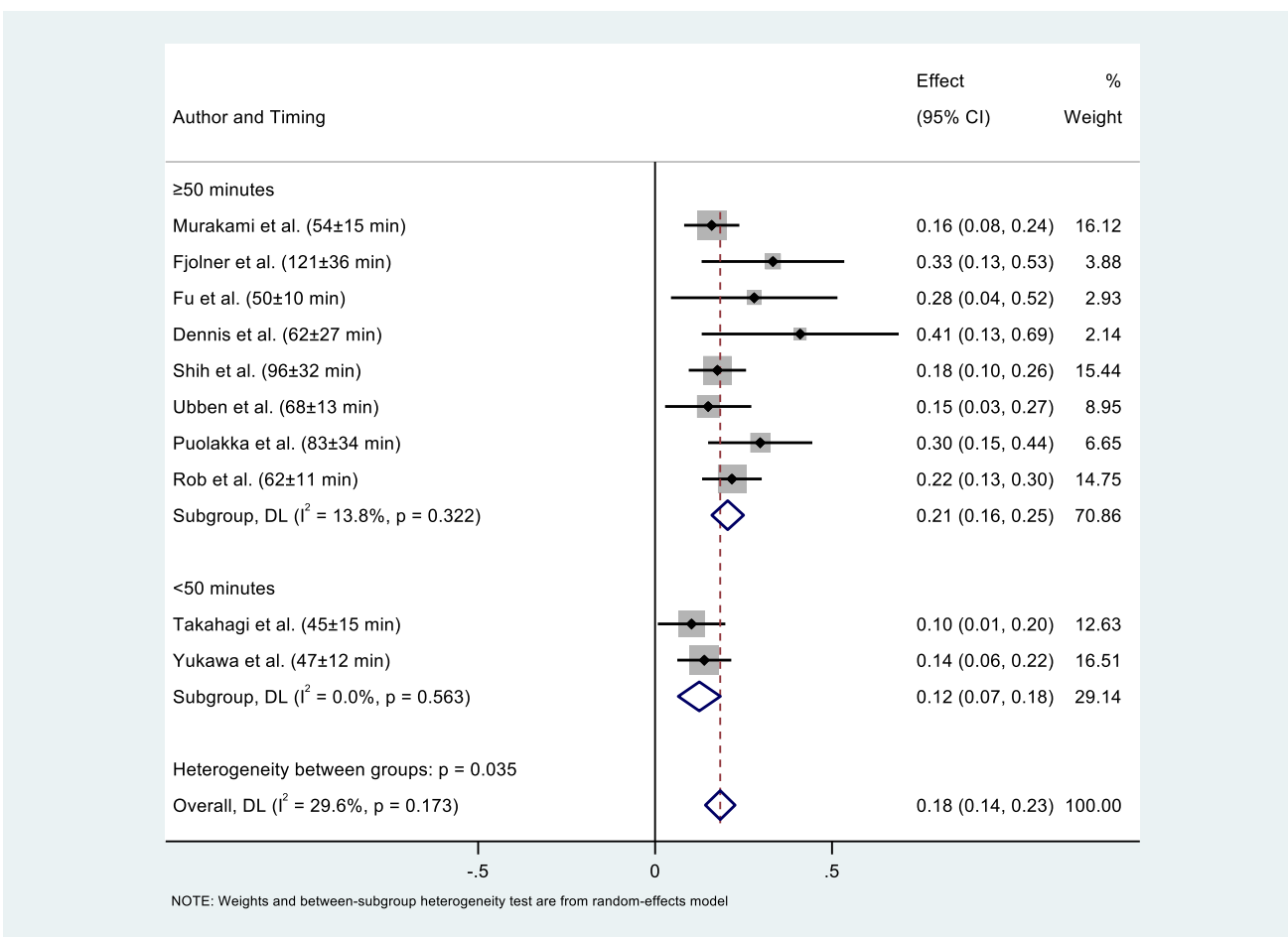

c) Forrest Plot of correlation between NFD with Survival at Discharge

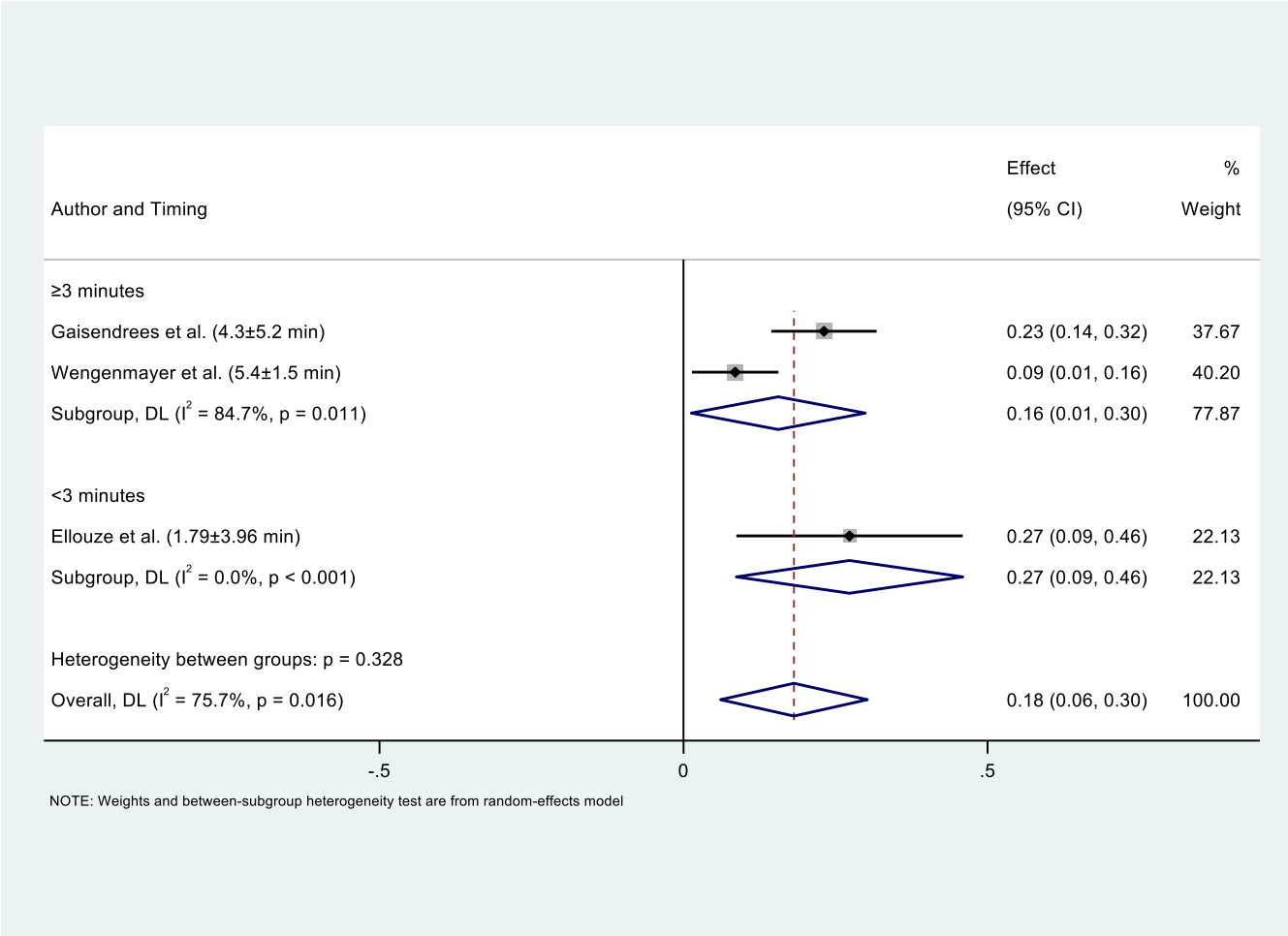

d) Forrest Plot of correlation between CA to ECMO Duration with Survival at Discharge

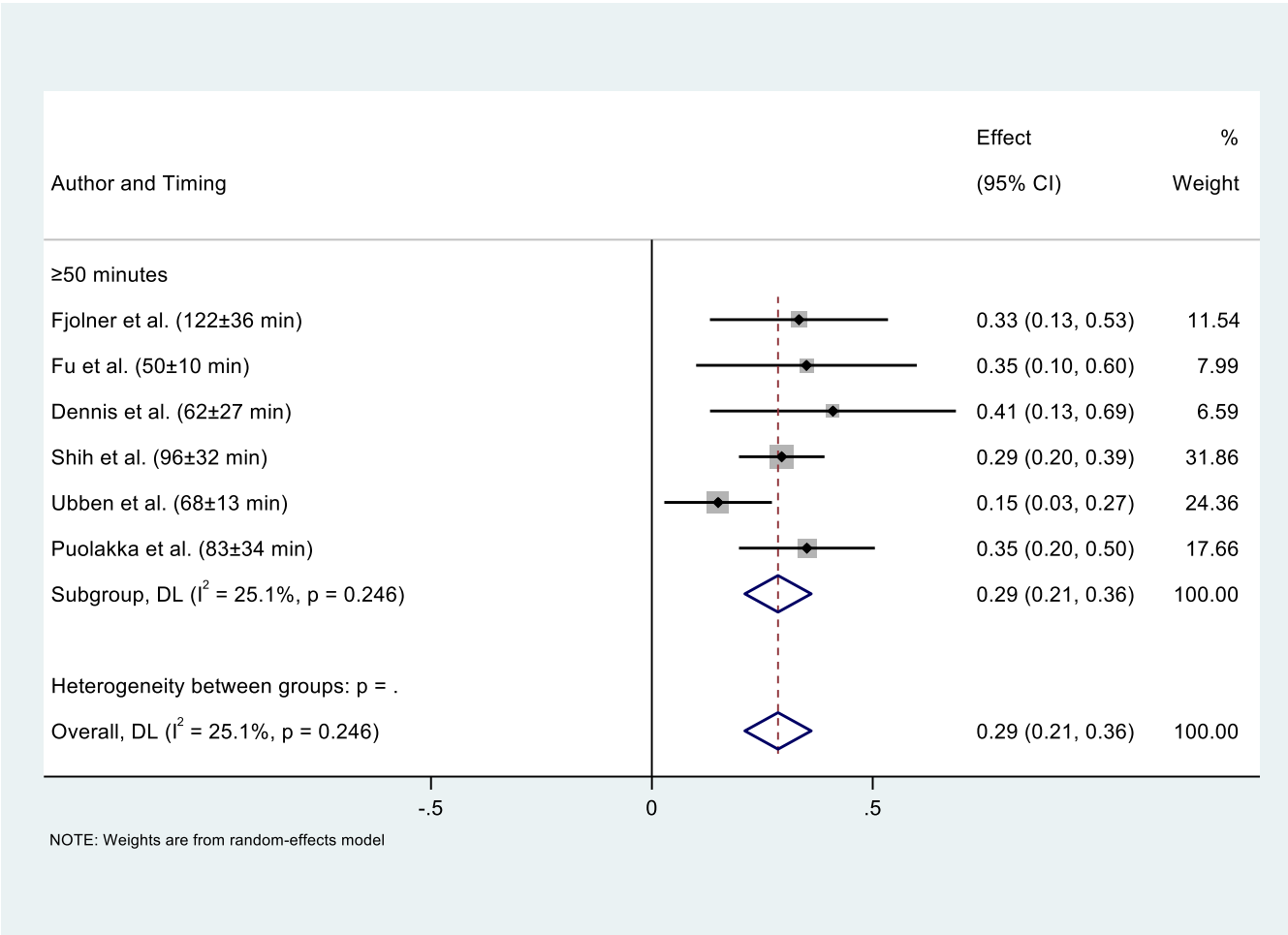

Supplementary Figure 3 – Forest Plots of IHCA Only Groups

a) Forrest Plot of correlation between NFD with Neurological Outcomes

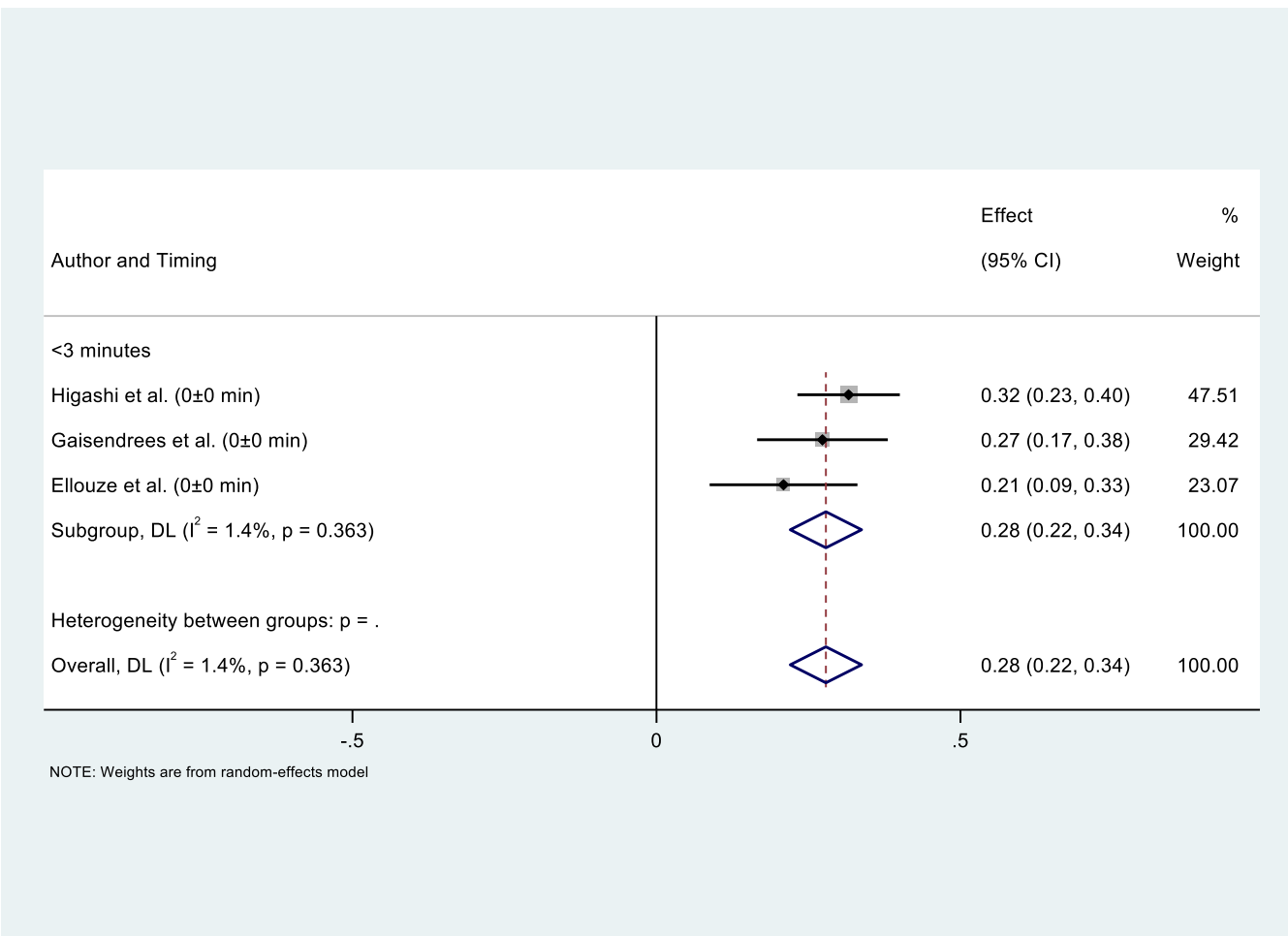

b) Forrest Plot of correlation between NFD with Survival at Discharge

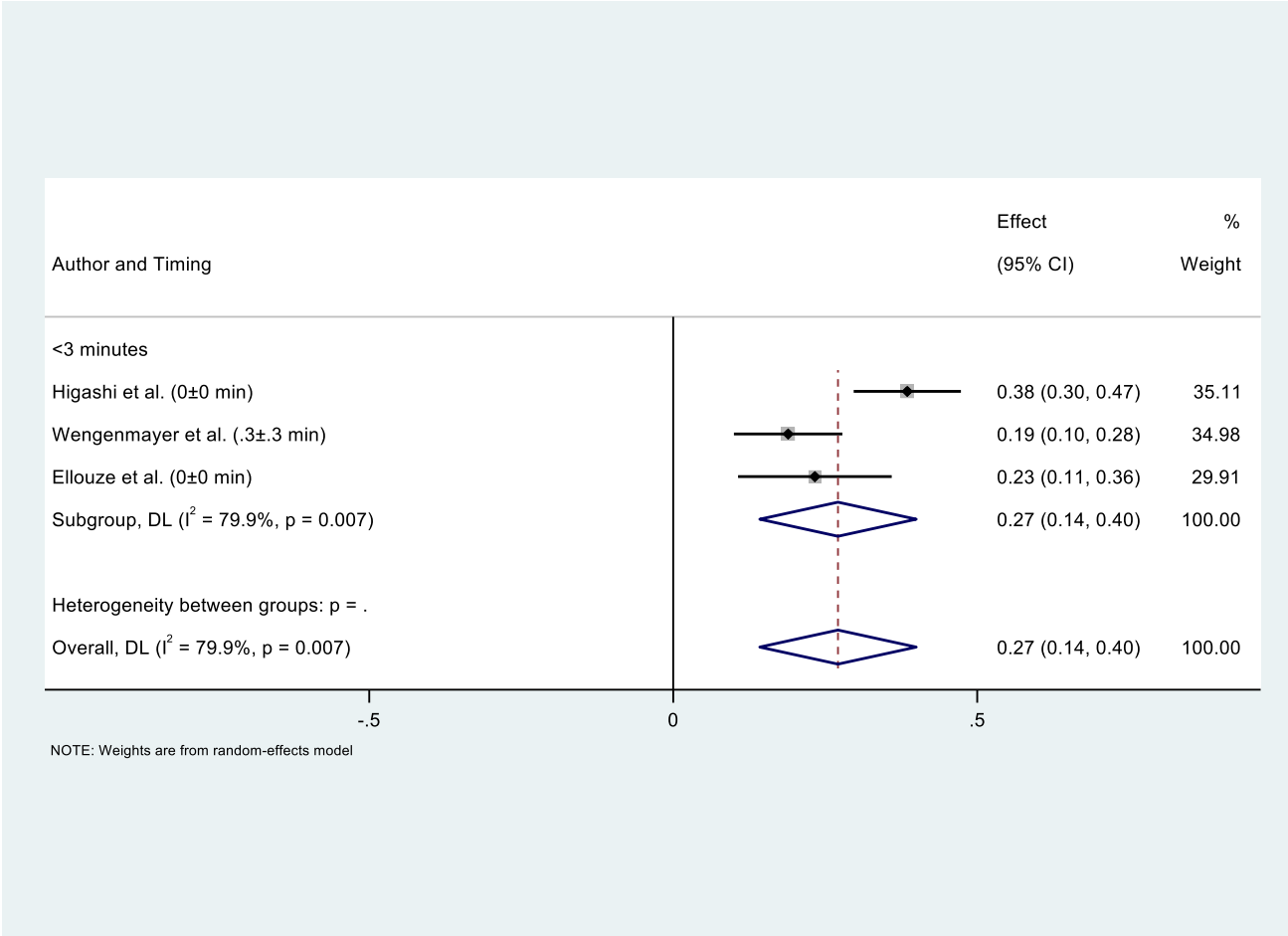

Supplementary Figure 4 – Risk of Bias and Quality Assessments

a) Risk of bias for non-randomized studies

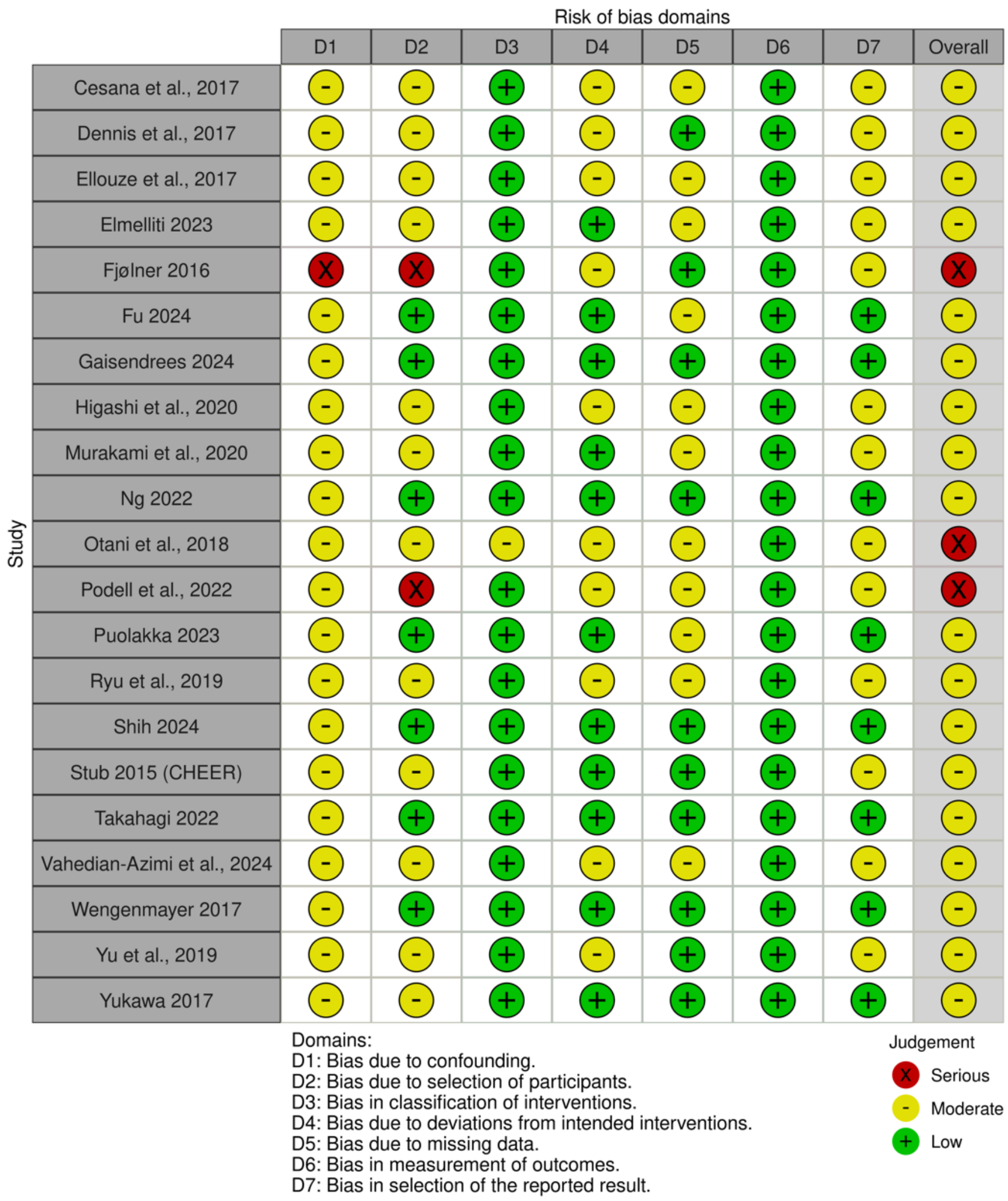

b) Risk of bias for non-randomized studies

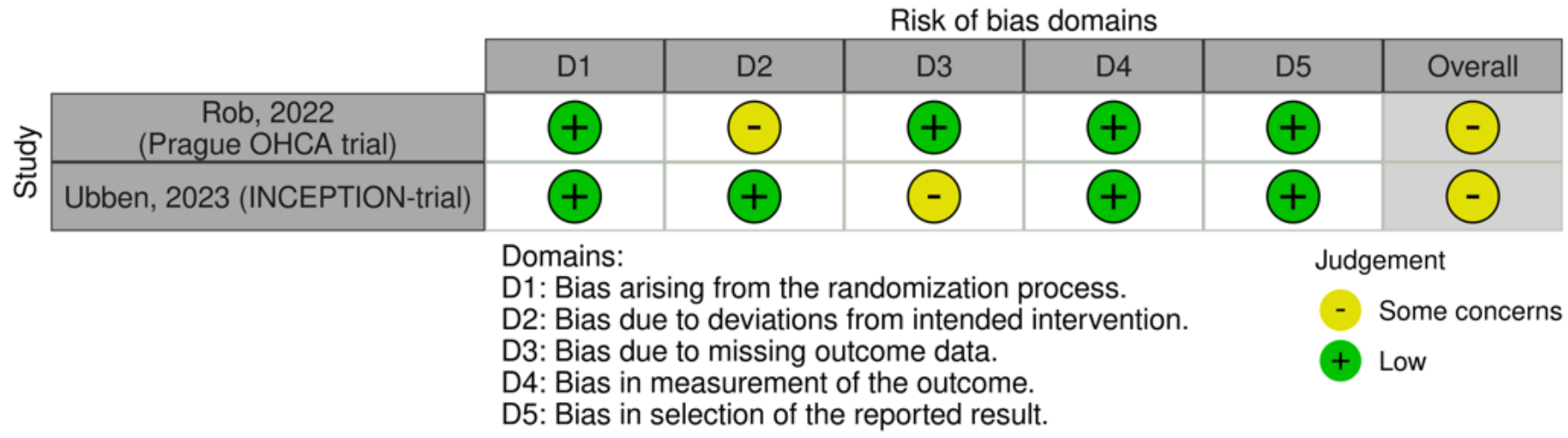

Supplement: Supplementary file 1 [file jccm-2026-0049-supplementary.pdf]
